# In silico SNP prediction of selected protein orthologues in insect models for Alzheimer's, Parkinson's, and Huntington’s diseases

**DOI:** 10.1038/s41598-023-46250-5

**Published:** 2023-11-03

**Authors:** Eshraka A. Al-Ayari, Magdi G. Shehata, Mohamed EL-Hadidi, Mona G. Shaalan

**Affiliations:** 1 Entomology Department, Faculty of Science, Ain Shams University, Cairo, Egypt; 2 Bioinformatics Group, Center for Informatics Sciences (CIS), School of Information Technology and Computer Science (ITCS) , Nile University, Giza, Egypt

**Keywords:** Gene expression, Proteomics, Entomology, Neurodegenerative diseases, Medical research

## Abstract

Alzheimer's, Parkinson’s, and Huntington’s are the most common neurodegenerative diseases that are incurable and affect the elderly population. Discovery of effective treatments for these diseases is often difficult, expensive, and serendipitous. Previous comparative studies on different model organisms have revealed that most animals share similar cellular and molecular characteristics. The meta-SNP tool includes four different integrated tools (SIFT, PANTHER, SNAP, and PhD-SNP) was used to identify non synonymous single nucleotide polymorphism (nsSNPs). Prediction of nsSNPs was conducted on three representative proteins for Alzheimer's, Parkinson’s, and Huntington’s diseases; APPl in *Drosophila melanogaster*, LRRK1 in *Aedes aegypti*, and VCPl in *Tribolium castaneum.* With the possibility of using insect models to investigate neurodegenerative diseases. We conclude from the protein comparative analysis between different insect models and nsSNP analyses that *D. melanogaster* is the best model for Alzheimer’s representing five nsSNPs of the 21 suggested mutations in the APPl protein. *Aedes aegypti* is the best model for Parkinson’s representing three nsSNPs in the LRRK1 protein. *Tribolium castaneum* is the best model for Huntington’s disease representing 13 SNPs of 37 suggested mutations in the VCPl protein. This study aimed to improve human neural health by identifying the best insect to model Alzheimer's, Parkinson’s, and Huntington’s.

## Introduction

Neurodegenerative diseases (NDs) are neurological disorders caused by progressive decline in brain function resulting from gradual neuronal death^1^. They are incurable and mostly affect the elderly population. Their incurability refers to the neural death which is the main cause of these diseases, and the late diagnosis where most symptoms appear in late stages of the diseases. The prevalence of age-related neurodegenerative diseases is increasing with age worldwide. The most recognized NDs were Alzheimer's disease (AD), Parkinson's disease (PD), and Huntington's disease (HD) respectively^2^ (Fig. S1). As they are not curable, their symptoms appear in late stages and lead to death. They negatively affect the quality of life of patients and their families both socially and economically^3^. Most NDs result from a combination of genetic and environmental factors, such as PD and AD, whereas others are caused by inherited mutant genes, such as HD.

Insects are suggested to serve as research model organisms because of their easy handling, small in size, small rearing places, relatively low rearing cost, short life cycles, high fecundity, rapid and simple gene manipulation, and fewer ethical permissions compared to vertebrate models^4,5^. The genomes of different model organisms have been sequenced in parallel with the human genomes, starting with *Drosophila melanogaster*^6^. The availability of multiple insect genomes creates an outstanding potential for comparative genomics among insects and between insects and humans. These comparative studies provide an effective tool for investigating human gene function compared to model insects^7,8^ Table S1. Many insect genes share common ancestry and function with human genes^9^. Decision-making centres in the brains of insects and mammals share many similarities in physiology although they have evolved independently^10^. The central complex in insects and the basal ganglia in vertebrates are similar in the maintenance of behavioural actions^11^. The hippocampi of vertebrates and mushroom bodies of arthropods were also similar in learning and memory (Fig. S2)^12^. Furthermore, insects provide phenotypic characteristics representing different NDs^13,14^ as shown in Table S2^15^. The dysfunctional brains of insects enable us to learn more about human brain diseases. In the AD *Drosophila* model for example, appearance of degenerated neurons and signs of edema in the hippocampus improve our understanding about what is happening^16^. In PD *Bombyx mori* model, The *p*-translucent silkworm is caused by downregulation of the DJ-1 gene, resulting in an increase in the oxidative stress response of the body, which leads to oxidative damage to the nerves and tissues^17,18^.

Dysfunctional gene behaviour is commonly caused by mutations that are primarily responsible for the development of illnesses. Many disease-causing mutations have been identified in the genome, around 0.5 million are SNPs^19^. This means that one base is replaced by one other base. Such mutations may involve synonymous and non-synonymous single nucleotide variants (SNVs) or SNPs that may fall within coding sequences of genes, non-coding regions of genes, or intergenic regions^20^. SNPs play a significant role and increase the susceptibility toward many diseases. Synonymous SNPs (sSNPs) in coding regions have no effect on translated proteins^21^. However, they can also affect mRNA stability and translation rate. Nonsynonymous SNPs (nsSNPs), which cause amino acid substitutions, have a direct impact on protein structure and function. SNPs in non-coding regions may affect gene splicing and other biological processes such as RNA degradation and transcription^22^.

Computational tools are used to predict the effects of mutations on protein function and structure. They are important for the analysis of SNVs and their prioritisation for experimental characterization. Using a *sequence homology algorithm,* computational tools can identify mutations that are significantly pathogenic based on their alignment with known pathogenic mutations as in SIFT, and PANTHER tools^23,24^. Other computational tools utilise *artificial neural networks*, and support vector machines to classify the nsSNVs into diseased or neutral substitutions as SNAP, and PhD-SNP tools^25,26^. *Consensus-based approaches* tool that integrate multiple algorithms to determine the pathogenicity of nsSNPs as Meta-SNP tool, that combine (SIFT, PANTHER, SNAP, and PhD-SNP)^27^.

**Figure 1:**
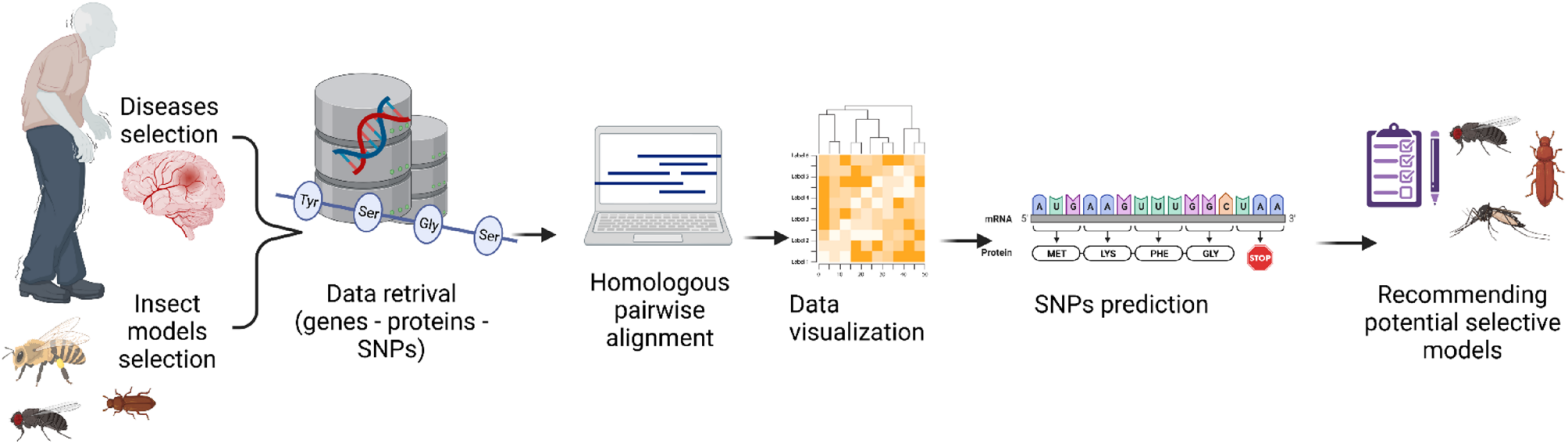
The sequence of the performed analyses.

Proving a causal link between a gene and disease is expensive and time-consuming. Therefore, the comprehensive prioritisation of candidate SNPs and determination of the best model to simulate the disease before experimental testing drastically reduces the associated costs, saves time, and accelerates the process of drug discovery as shown in (Fig. 1). Our aim is to highlight the best insect to model Alzheimer's, Parkinson's, and Huntington’s diseases; even in case of the selection of a specific protein to be deeply studied or for overall simulating one of the diseases. Based on the predicted nsSNPs in insect proteins compared to human proteins, simulating diseases’ mechanisms and pathways will be easier and will help improve drug discovery of these NDs.

## Materials and methods

This research paper was approved by the research ethics committee from the Faculty of Science, Ain Shams University (ASU-SCI/ENTO/2023/8/1).

### Dataset retrieval

#### All retrieved data are from publicly available databases

- The information of the genes of interest was retrieved based on the most influential disease-causing genes from the literature “GeneReviews®—NCBI Bookshelf'' (https://www.ncbi.nlm.nih.gov/books/)^28^, KEGG DISEASE Database (https://www.kegg.jp/kegg/disease/)^29–31^ (Figs. S3, S4, and S5), and other manual searches using keywords “Alzheimer genes, Parkinson genes, Huntington genes” on Pubmed and Google Scholar. Selected genes included 10 genes for AD, 13 genes for PD, and 10 genes for HD, as shown in Table S3 (accessed: February, 2023).
  AD: APP, COL25A1, GRN, HDAC6, MAPT, Nep2, PSN-1, PSN-2, RAC1, and SORL1.
  PD: PRKN, Pink1, DJ-1, GAK, VPS35, UCHL1, EIF4G1, ATP13A2, GIGYF2, HTRA2, PLA2G6, FBXO7, and LRRK2.
  HD: HTT, DMBK, GRIK2, VCP, VPS13A, ATXN1, ALS, MJD, UBQLN2, and CACNA1A.
- Protein sequences were retrieved from NCBI using the Protein database (https://www.ncbi.nlm.nih.gov/protein/), Blastp tool (https://blast.ncbi.nlm.nih.gov/Blast.cgi)^32^, and genes with similar protein architectures were searched using NCBI's SPARCLE (https://www.ncbi.nlm.nih.gov/protfam/) as a resource of the sequential arrangement of CD domains.
- Human nsSNPs within the coding region were selected for the APP, LRRK2, and VCP genes (each as a representative gene for Alzheimer’s, Parkinson’s, and Huntington’s diseases, respectively). Polymorphism data were retrieved from the dbSNP-SNP database of NCBI (https://www.ncbi.nlm.nih.gov/snp/) with selection criteria (pathogenic and somatic).

### Model selection

The selection of insect models was based on some criteria; these eight selected insect models were taxonomized in four different insect orders (the four largest insect orders)^33^. Selected insect models are the most common, fully sequenced insects, and are the most representative species of their orders. According to their taxonomic classification; (1) Order Diptera: *Drosophila melanogaster* (*D. melanogaster*)*, Musca domestica* (*M. domestica*)*, Anopheles gambiae* (*An. gambiae*)*, and Aedes aegypti* (*A. aegypti*), (2) Order Hymenoptera*: Apis mellifera (A. mellifera),* (3) Order Coleoptera: *Tribolium castaneum (T. castaneum),* (4) Order Lepidoptera: *Bombyx mori (B. mori),* and *Galleria mellonella (G. mellonella)*. The insect models were confirmed based on InsectBase (http://v2.insect-genome.com/Classify/Model%20Organism)^34^. In addition to the *Mus musculus* as a transition mammalian model and a distant from insect models.

### Bioinformatics analyses

1. Pairwise alignment was performed to detect protein homology and identify query coverage and percentage of protein identity. Alignment was performed between each protein in *H. sapiens* against its homolog in the selected model organisms using BLASTP (Basic Local Alignment Search Tool for protein) with default parameters from NCBI^32^, except (GRN and NEP2) alignments were performed against *D. melanogaster* because they showed no alignment against *H. sapiens.*
2. In Silico SNP prediction of disease-causing variants was performed using the publicly available tool Meta-SNP (meta-predictor of disease-causing variants)^27,35,36^. This tool permits the detection of disease-associated nsSNVs for both well-identified and predicted amino acid sequences (SNPs based on dbSNP of humans) (accessed 22 June, 2023). This approach is characterised by other methods by integrating four existing methods: PANTHER, PhD-SNP, SIFT, and SNAP with defined default threshold parameters PANTHER, PhD-SNP, and Meta-SNP: Between 0 and 1 (If > 0.5, mutation is predicted Disease), SIFT: Positive Value (If > 0.05 mutation is predicted Neutral), SNAP: Output normalised between 0 and 1 (If > 0.5, mutation is predicted Disease).
  - A local alignment search was performed between the substituted amino acid in the human protein and its homolog protein in the selected insect model using BLASTP and manual search, depending on finding the best match using 5 aa before and 5 aa after the substituted amino acid to provide a proper short sequence needed to find the accurate position of required aa.
  - The matched amino acids and protein sequence were entered into the meta-SNP analysis tool to determine the probability of causing disease for amino acid substitutions according to the human nsSNPs.

## Results

### Pairwise alignment

Pairwise alignment using blastp with default parameters was conducted for each selected insect protein against its homolog in humans, except for GRN and NEP2, where pairwise alignment was conducted against the fruit fly (Tables S4, S5, and S6). A sharp cut-off value for homology, 75% query coverage, and 30% protein identity was applied to filter the results with the more meaningful values^37–39^ .

**Figure 2:**
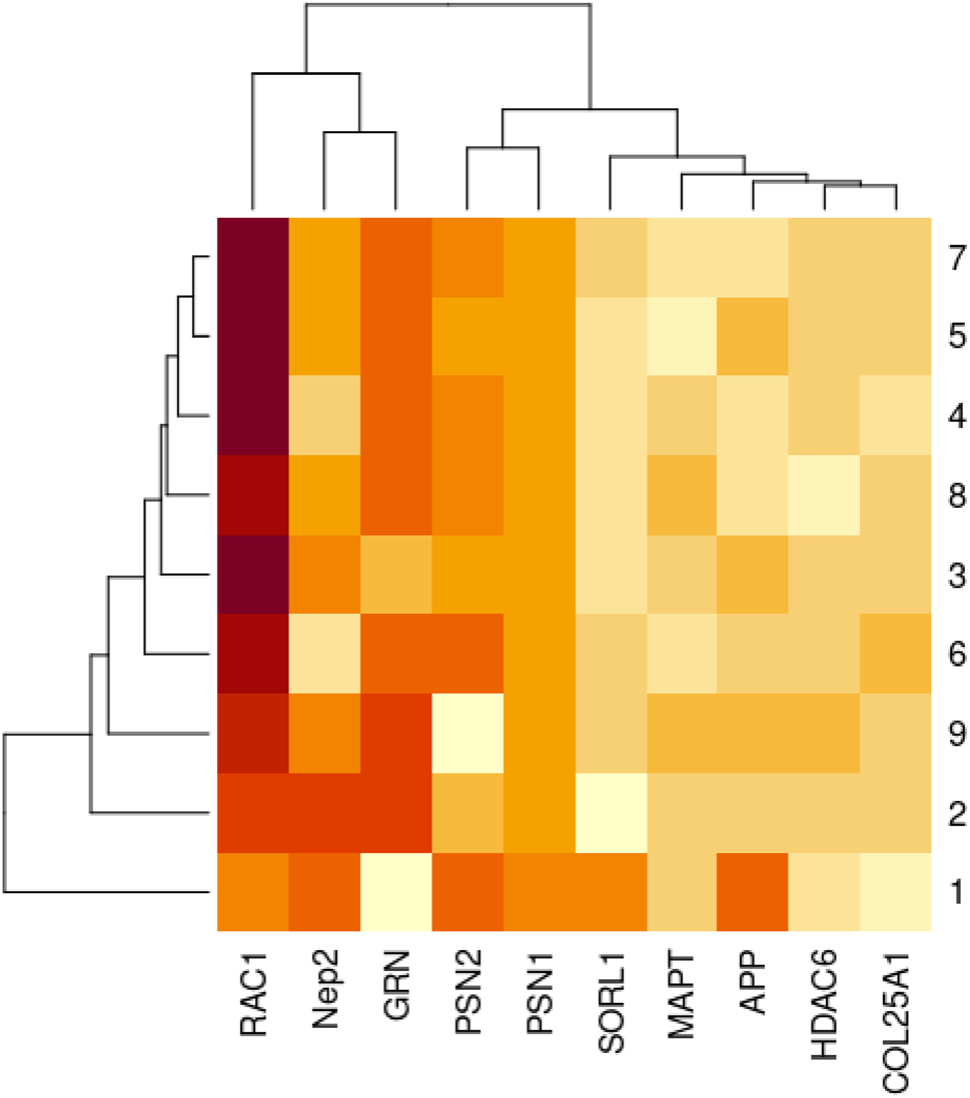
The heatmap shows the percentage of protein identity for AD proteins between different insect models, 1 *Mus musculus, 2 Drosophila melanogaster, 3 Apis mellifera, 4 Tribolium castaneum, 5 Bombyx mori, 6 Musca domestica, 7 Galleria mellonella, 8 Anopheles gambiae, 9 Aedes aegypti.* Where deep colour refers to high protein identity and light colour refers to low protein identity. The heatmap was generated using RStudio version 2022.12.0 + 353.

The results showed that:

*For Alzheimer’s disease* Table 1: *A. mellifera* shows greater identity to *H. sapiens* than *D. melanogaster* for APP protein*. Musca domestica* has more identity with *H. sapiens* than *D. melanogaster* for COL25A1 protein. *Aedes aegypti is the nearest in identity* to *D. melanogaster* for the GRN protein. *Musca domestica, and A. aegypti show greater identity to H. sapiens* than *D. melanogaster for* HDAC6 protein. *Galleria mellonella* has the closest Tau/Mapt protein identity to *H. sapiens* beside *D. melanogaster. Aedes aegypti s*hows greater identity to *D. melanogaster* for the Nep2 protein. *Tribolium castaneum,* and *B. mori* have more identical Psn1 and Psn2 to *H. sapiens* than *D. melanogaster*. For RAC1 *T. castaneum*, *M. domestica,* and *G. mellonella* were more identical to *H. sapiens* than *D. melanogaster. In the absence of* SORL1 in *D. Melanogaster; A. mellifera, T. castaneum*, and *A. aegypti* showed a higher identity with *H. sapiens*. As shown in (Fig. 2).

**Table 1:** The protein identity percentages between human, *Mus musculus* and other selected insect models in AD, with cut off 75% of query coverage and 30% of protein identity besides the fruit fly as a reference insect model.

	APP				COL25A1				GRN				HDAC6				MAPT–Tau			
	ID	Query cover (%)	Length	Identity (%)	ID	Query cover (%)	Length	Identity (%)	ID	Query cover (%)	Length	Identity (%)	ID	Query cover (%)	Length	Identity (%)	ID	Query cover (%)	Length	Identity (%)
1	NP_001185753.1	100	751 aa	94.81	NP_001231881.1	96	645 aa	76.42	NP_032201.3	100	589 aa	75.21	NP_034543.3	99	1149 aa	79.31	NP_001390904.1	92	683 aa	83.33
2	NP_001245452.1	60	888 aa	36.27	NP_723044.1	**92**	1779 aa	39.12	NP_001138026.1	**100**	699 aa	*100.00*	NP_001162760.1	41	883 aa	35.00	NP_001287564.1	48	288 aa	*36.94*
3	XP_026299199.1	**76**	704 aa	*42.71*	XP_393523.5	76	1501 aa	38.57												
4					XP_008193734.1	77	1917 aa	35.12												
5					XP_037866540.1	86	1806 aa	40.81												
6					XP_005186422.1	**92**	1773 aa	*43.73*					XP_019892510.1	77	918 aa	35.34				
7					XP_026757358.2	79	1896 aa	41.51												
8					XP_318597.4	76	997 aa	41.11												
9					XP_021697121.1	78	777 aa	36.44					XP_021694948.1	79	1115 aa	*40.50*				

	Nep2				PSN-1				PSN-2				RAC1				SORL1			
	ID	Query cover (%)	Length	Identity (%)	ID	Query cover (%)	Length	Identity (%)	ID	Query cover (%)	Length	Identity (%)	ID	Query cover (%)	Length	Identity (%)	ID	Query cover (%)	Length	Identity (%)
1	NP_001344264.1	82	750 aa	95.45	NP_001349200.1	100	467 aa	92.72	NP_001122077.1	100	448 aa	95.98	NP_001334459.1	100	211 aa	91.00	NP_035566.2	100	2215 aa	93.18
2	NP_001246914.1	**100**	774 aa	*100*	NP_001137988.1	85	508 aa	55.66	NP_524184.1	87	541 aa	52.70	NP_001261247.1	**100**	192 aa	91.67				
3	XP_006566920.1	99	764 aa	57.22	XP_006564247.2	96	500 aa	52.73	XP_006564247.2	**99**	500 aa	51.49	XP_623951.1	**100**	192 aa	91.15	XP_006567452.1	**95**	2153 aa	32.91
4	XP_971821.3	99	786 aa	38.56	XP_967139.2	96	463 aa	*55.94*	XP_967139.2	98	463 aa	56.75	XP_968397.1	**100**	192 aa	93.23	XP_008191758.1	**95**	2121 aa	33.39
5	XP_037872516.1	98	759 aa	54.16	XP_004924557.1	**97**	482 aa	55.22	XP_004924557.1	**99**	482 aa	51.44	XP_004932553.1	**100**	193 aa	90.16				
6					XP_011294721.1	94	511 aa	51.92	XP_011294718.1	77	523 aa	*63.92*	XP_005176777.1	**100**	192 aa	92.71				
7	XP_026758864.1	99	758 aa	54.66	XP_026760544.1	95	501 aa	52.61	XP_026760544.1	86	501 aa	57.10	XP_026751245.1	**100**	193 aa	91.71				
8	XP_321277.5	99	767 aa	59.72	XP_311942.4	91	523 aa	55.08	XP_311942.4	76	523 aa	63.64	XP_315449.4	93	192 aa	*94.41*				
9	XP_021705640.1	**100**	798 aa	58.90	XP_021693761.1	97	557 aa	50.77					XP_021696819.1	**100**	192 aa	91.67	XP_021706160.1	**95**	2188 aa	*36.28*

Highest protein identities are highlighted in italic and highest query coverages are highlighted in bold. 1 *Mus musculus, 2 Drosophila melanogaster, 3 Apis mellifera, 4 Tribolium castaneum, 5 Bombyx mori, 6 Musca domestica, 7 Galleria mellonella, 8 Anopheles gambiae, 9 Aedes aegypti.*

**Figure 3:**
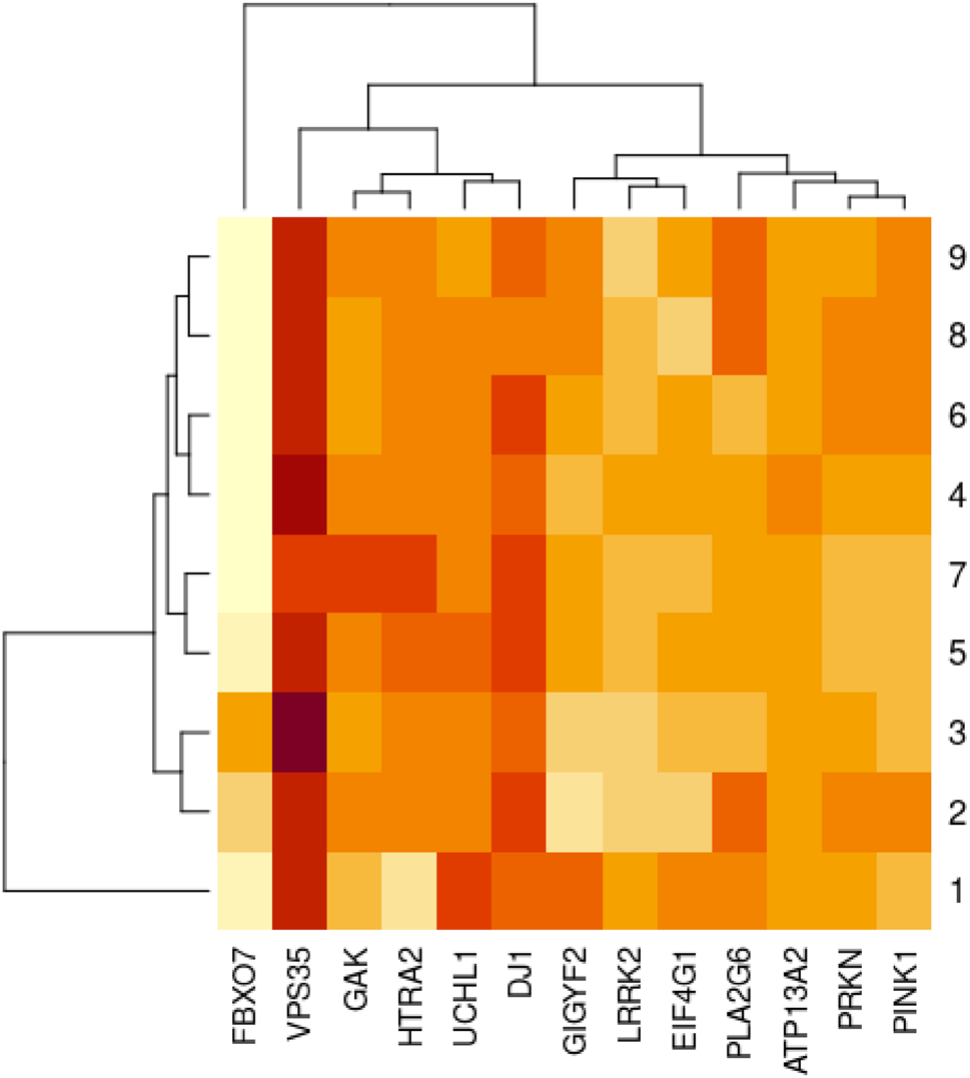
The heatmap shows the percentage of protein identity for PD proteins between different insect models, 1 *Mus musculus, 2 Drosophila melanogaster, 3 Apis mellifera, 4 Tribolium castaneum, 5 Bombyx mori, 6 Musca domestica, 7 Galleria mellonella, 8 Anopheles gambiae, 9 Aedes aegypti.* Where deep colour refers to high protein identity and light colour refers to low protein identity. The heatmap was generated using RStudio version 2022.12.0 + 353.

*For Parkinson’s disease* Table 2: *M. domestica* and *A. gambiae* show better protein identity to *H. sapiens* than *D. melanogaster* for DJ-1 protein. *Tribolium castaneum* has greater GAK, HTRA2, LRRK2, and EIF4G1 protein identity to *H. sapiens* than *D. melanogaster.* In the case of VPS35, *A. mellifera* showed the highest protein identity with *H. sapiens. B. mori* showed higher UCHL1 protein identity than *D. melanogaster*. For *A. aegypti* and *G. mellonella,* ATP13A2 protein showed more identity to *H. sapiens* than *D. melanogaster.* In addition, *G. mellonella* has a better GIGYF2 protein identity with *H. sapiens* than *D. melanogaster. A. gambiae* showed greater identity to *H. sapiens* than *D. melanogaster* for the PLA2G6 protein. As shown in Fig. 3.

**Table 2:** The protein identity percentages between human, Mus musculus and other selected insect models in PD, with cut off 75% of query coverage and 30% of protein identity besides the fruit fly as a reference insect model.

	PRKN–PARKIN–PARK2				Pink1–PARK6				PARK7–DJ-1				GAK				PARK17–VPS35			
	ID	Query cover (%)	Length	Identity (%)	ID	Query cover (%)	Length	Identity (%)	ID	Query cover (%)	Length	Identity (%)	ID	Query cover (%)	Length	Identity (%)	ID	Query cover (%)	Length	Identity (%)
1	NP_001390399.1	100.00	465 aa	83.87	NP_081156.2	99	580 aa	81.55	NP_065594.2	100	189 aa	91.53	NP_001346853.1	87	1144 aa	81.06	NP_075373.1	100	796 aa	99.37
2	NP_730600.1	**99.00**	482 aa	*42.19*	NP_001027049.1	57	721 aa	42.99	NP_651825.4	97	187 aa	52.69	NP_001262243.1	74	1153 aa	41.79	NP_611651.4	97	803 aa	61.49
3	XP_396426.5	**99.00**	498 aa	41.14	XP_006563863.1	**93**	614 aa	34.77	XP_006563123.1	95	222 aa	47.51					XP_392327.4	98	1149 aa	*69.51*
4	XP_969040.2	92.00	440 aa	37.41	XP_968367.1	81	570 aa	36.74	XP_973301.2	97	213 aa	50.27	XP_967193.1	80	1123 aa	*43.62*	XP_967674.1	**99**	801 aa	67.42
5									NP_001232899.1	97	190 aa	51.87					XP_004926171.2	**99**	808 aa	62.47
6	XP_005175480.2	**99.00**	479 aa	42.07					XP_005186506.1	97	208 aa	*55.91*	XP_011293498.2	**84**	1269 aa	39.64	XP_005182752.1	98	819 aa	61.10
7																	XP_031768069.1	**99**	764 aa	54.46
8	XP_316606.2	**99.00**	489 aa	41.44					XP_311236.5	**98**	212 aa	40.95					XP_308188.4	98	810 aa	63.42
9	XP_001648798.2	**99.00**	483 aa	40.25	XP_021704788.1	76	708 aa	*43.79*	XP_001648396.2	94	186 aa	50.00					XP_021696035.1	98	805 aa	64.74

	PARK5–UCHL1				PARK18–EIF4G1				PARK9–ATP13A2				PARK11–GIGYF2				PARK13–HTRA2			
	ID	Query cover (%)	Length	Identity (%)	ID	Query cover (%)	Length	Identity (%)	ID	Query cover (%)	Length	Identity (%)	ID	Query cover (%)	Length	Identity (%)	ID	Query cover (%)	Length	Identity (%)
1	NP_035800.2	100	223 aa	95.52	NP_666053.2	100	1600 aa	90.57	NP_001366548.1	100	1168 aa	85.34	NP_666224.3	100	1291 aa	*93.49*	NP_062726.3	100	458 aa	76.50
2	NP_001188681.1	**99**	227 aa	45.13	NP_001096852.1	**62**	1919 aa	*27.76*	NP_001096849.1	**96**	1451 aa	36.42	NP_651950.3	57	1567 aa	24.17	NP_001262565.1	66	422 aa	*44.58*
3	XP_392902.1	97	234 aa	42.67					XP_006564975.1	95	1435 aa	39.90								
4	XP_966886.1	**99**	232 aa	45.89					XP_015834823.1	90	1106 aa	*42.96*					XP_008190859.1	**87**	402 aa	40.38
5	XP_004932329.1	98	230 aa	*49.33*					XP_037876978.1	**96**	1190 aa	34.95								
6	XP_005187155.1	97	228 aa	45.05					XP_005188409.1	95	1602 aa	37.17								
7	XP_026758708.1	98	230 aa	46.22					XP_026760352.1	**96**	1219 aa	36.68								
8	XP_320415.2	96	228 aa	45.70					XP_309375.4	93	1278 aa	37.59								
9	XP_001663268.1	96	228 aa	41.63					XP_001659588.2	**96**	1322 aa	37.15								

	PARK14–PLA2G6/ revised				PARK15–FBXO7/revised				LRRK2–PARK8/revised											
	ID	Query cover (%)	Length	Identity (%)	ID	Query cover (%)	Length	Identity (%)	ID	Query cover (%)	Length	Identity (%)								
1	NP_001185952.1	100	807 aa	89.71	NP_694875.2	100	523 aa	72.90	NP_080006.3	100	2527 aa	86.60								
2	NP_648366.2	90	877 aa	50.89	NP_001189211.1	**20**	442 aa	*27.42*	NP_001262772.1	42	2513 aa	*28.47*								
3	XP_006565723.1	97	798 aa	36.21																
4	XP_008196222.1	97	794 aa	36.49																
5	XP_004931589.2	97	808 aa	34.90																
6	XP_019895588.1	94	889 aa	30.76																
7	XP_026762452.1	**98**	807 aa	34.51																
8	XP_317997.3	92	893 aa	*52.78*																
9	XP_001656280.2	89	901 aa	52.16																

Highest protein identities are highlighted in italic and highest query coverages are highlighted in bold. 1 *Mus musculus, 2 Drosophila melanogaster, 3 Apis mellifera, 4 Tribolium castaneum, 5 Bombyx mori, 6 Musca domestica, 7 Galleria mellonella, 8 Anopheles gambiae, 9 Aedes aegypti.*

**Figure 4:**
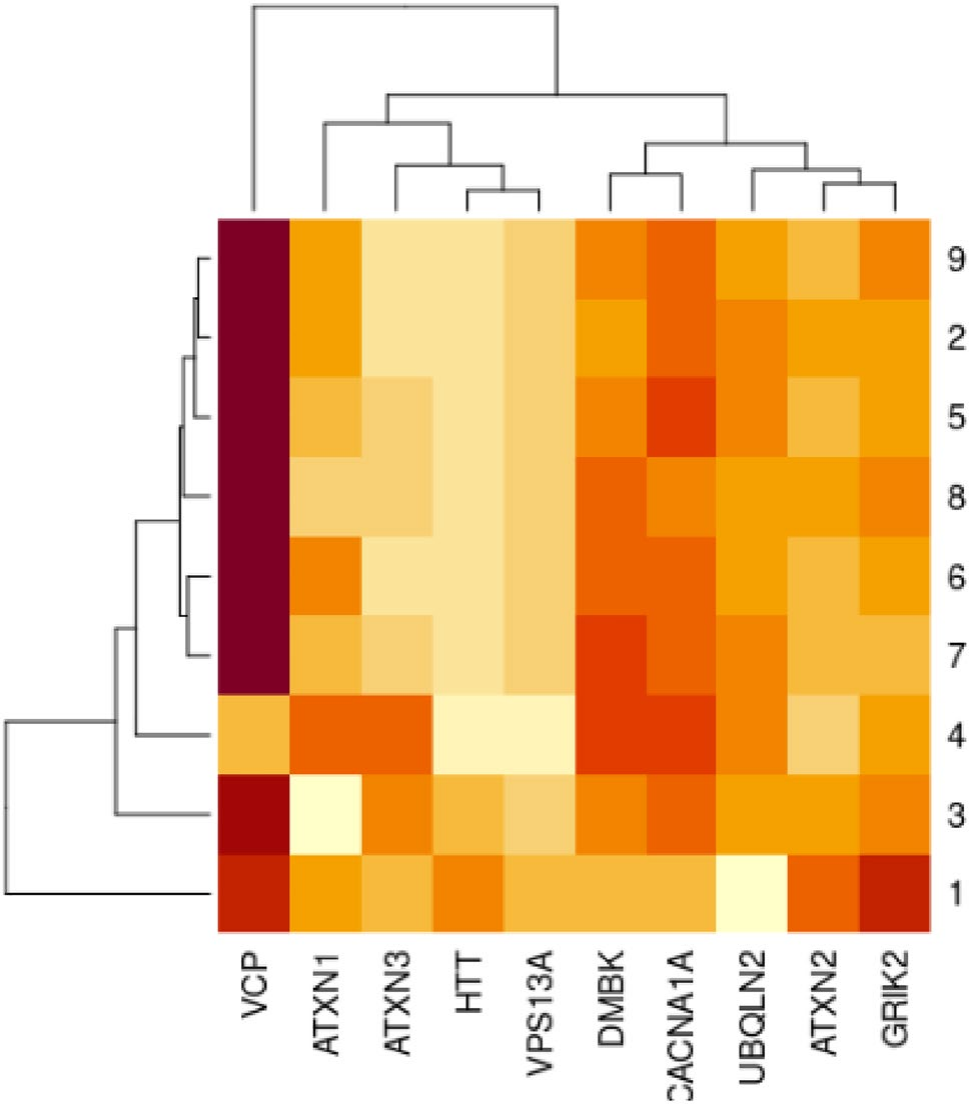
The heat map shows the percentage of protein identity for HD proteins between different insect models, 1 *Mus musculus, 2 Drosophila melanogaster, 3 Apis mellifera, 4 Tribolium castaneum, 5 Bombyx mori, 6 Musca domestica, 7 Galleria mellonella, 8 Anopheles gambiae, 9 Aedes aegypti.* Where deep colour refers to high protein identity and light colour refers to low protein identity. The heatmap was generated using RStudio version 2022.12.0 + 353.

*For Huntington’s disease* Table 3: *A. mellifera* has higher HTT, UBQLN2, and DMBK protein identity to *H. sapiens* than *D. melanogaster. B. mori* has better GRIK2 and ATXN1 protein identity to *H. sapiens* than *D. melanogaster. Apis mellifera, A. gambiae, and A. aegypti* were closer to *H. sapiens* than *D. melanogaster* for the VCP protein. *Tribolium castaneum* has greater VPS13A and ATXN3 protein identity to *H. sapiens* than *D. melanogaster. A. gambiae* showed better CACNA1A protein identity with *H. sapiens* than *D. melanogaster.* As shown in Fig. 4.

**Table 3:** The protein identity percentages between human , Mus musculus and other selected insect models in HD, with cut off 75% of query coverage and 30% of protein identity besides the fruit fly as a reference insect model.

	HTT				DMBK				GRIK2				VCP				VPS13A			
	ID	Query cover (%)	Length	Identity (%)	ID	Query cover (%)	Length	Identity (%)	ID	Query cover (%)	Length	Identity (%)	ID	Query cover (%)	Length	Identity (%)	ID	Query cover (%)	Length	Identity (%)
1	NP_034544.1	97	3120 aa	91.19	NP_115794.1	89	631 aa	85.49	NP_001104738.2	100	908 aa	99.01	NP_033529.3	100.00	806 aa	100.00	NP_766616.2	100	3166 aa	84.34
2	NP_001263041.1	47	3583 aa	26.59	NP_523837.2	72	1637 aa	44.36	NP_651941.2	**94**	910 aa	44.87	NP_477369.1	**99.00**	801 aa	83.19	NP_001260781.1	93	3321 aa	30.96
3	XP_001122101.2	88	2887 aa	*32.81*	XP_016766179.1	**76**	1860 aa	*47.43*	XP_026301856.1	90	867 aa	48.46	XP_006563745.1	**99.00**	800 aa	84.02	XP_026298325.1	**99**	3245 aa	31.19
4									XP_015840808.1	91	851 aa	48.04					XP_015838119.1	**99**	3218 aa	31.43
5									XP_021207900.1	**94**	911 aa	48.00	NP_001037003.1	**99.00**	805 aa	83.98	XP_037868684.1	**99**	3259 aa	30.47
6									XP_019891420.1	93	854 aa	46.95	XP_005182894.1	**99.00**	801 aa	83.56	XP_005179023.1	92	3382 aa	31.28
7									XP_031766702.1	86	917 aa	39.23	XP_026762943.1	95.00	805 aa	*86.05*	XP_031767541.1	87	3043 aa	*31.90*
8									XP_003437104.1	90	888 aa	*48.98*	XP_315644.3	**99.00**	804 aa	84.33	XP_558472.3	98	3290 aa	30.21
9									XP_021697662.1	92	924 aa	48.30	XP_001654680.1	**99.00**	803 aa	84.68	XP_021697209.1	98	3363 aa	30.66

	SCA2–ALS				SCA3–MJD–ATXN3				SCA1/ATXN1				UBQLN2				CACNA1A			
	ID	Query cover (%)	Length	Identity (%)	ID	Query cover (%)	Length	Identity (%)	ID	Query cover (%)	Length	Identity (%)	ID	Query cover (%)	Length	Identity (%)	ID	Query cover (%)	Length	Identity (%)
1	NP_033151.2	92	1286 aa	94.02	NP_083981.2	100	355 aa	85.05	NP_001186233.1	100	791 aa	86.89	NP_081118.4	100	582 aa	72.64	NP_031604.3	100	2368 aa	85.82
2	NP_732033.1	**35**	1084 aa	*44.05*	NP_572303.4	47	221 aa	*26.67*	NP_572356.1	13	230 aa	*44.25*	NP_001285457.1	70	547 aa	49.77	NP_996416.1	65	1851 aa	*59.62*
3													XP_006562529.1	**94**	529 aa	45.82				
4													XP_008200719.1	83	537 aa	*49.78*				
5																				
6													XP_005189617.1	**94**	523 aa	45.41				
7													XP_026749925.1	78	515 aa	49.53				
8																	XP_003436163.1	**87**	1875 aa	50.81
9													XP_001652587.1	80	505 aa	47.76				

Highest protein identities are highlighted in italic and highest query coverages are highlighted in bold. 1 *Mus musculus, 2 Drosophila melanogaster, 3 Apis mellifera, 4 Tribolium castaneum, 5 Bombyx mori, 6 Musca domestica, 7 Galleria mellonella, 8 Anopheles gambiae, 9 Aedes aegypti.*

### In Silico nsSNPs prediction

In Silico nsSNPs prediction is performed using five integrated tools (SIFT, PANTHER, SNAP, PhD-SNP, and Meta-SNP).

Polymorphism data for APP (NP_001191231.1), LRRK2 (NP_940980.4), and VCP (NP_009057.1) proteins were retrieved from the NCBI dbSNP database as a publicly available database. Accordingly, APP was found to contain four missense SNPs in its coding regions. The LRRK2 gene was found to have one missense SNP in its coding region, and the VCP gene was found to have five missense SNPs in its coding region, but two of them rs779834525, and rs1420316004, were related to the FANCG gene and not VCP.

*For Alzheimer’s disease*, SNP analysis was performed on *D. melanogaster* App-like protein (NP_001245452.1) as a homolog of *H. sapiens* APP with 36.27% protein identity*,* using reference human SNPs rs63750264 (V > L,F,I), rs63750643 (T > A), rs63750671 (A > G), and rs193922916 (A > V,G).

1. In rs63750264, Val680Phe or Val680Leu, or Val680Ile in humans matches Val at positions 94, 863, and 869 in *D. melanogaster.*
2. In rs63750643, Thr677Ala in humans matches the Thr at position 742 in *D. melanogaster.*
3. In rs63750671, human Ala655Gly matches Ala at positions 180, 802, and 820 in *D. melanogaster.*
4. In rs193922916, Ala636Val or Ala636Gly in humans matches Ala at positions 138, 246, 713, and 758 in *D. melanogaster.*

Prediction using the SIFT, PANTHER, SNAP, PhD-SNP, and Meta-SNP tools results from 21 input-suggested mutations. Eleven mutations were predicted, Five of the 11 mutations showed deleterious or diseased points Table 4. Mutations V94F, V94L, A758G, A758V, and A820G are thought to be pathogenic in the AD *D. melanogaster* model according to PANTHER, Phd-SNP, and Meta-SNP while SIFT and SNAP couldn’t identify the effects of nsSNPs. In spite of the fact that A820G has the highest reliability index.

**Table 4:** Predicted nsSNPs V94F, V94L, A758G, A758V, and A820G in *D. melanogaster* Appl protein. *Italic* for diseased effect and **Bold** for neutral effect.

Mutation	PANTHER	Phd-SNP	SIFT	SNAP	Meta-SNP	RI
V94F	**Neutral** **0.496**	*Disease* *0.726*	NA _	NA _	*Disease* *0.502*	0
V94I	**Neutral** **0.171**	**Neutral** **0.292**	NA _	NA _	**Neutral** **0.302**	4
V94L	**Neutral** **0.225**	*Disease* *0.523*	NA _	NA _	**Neutral** **0.475**	1
A180G	**Neutral** **0.373**	**Neutral** **0.068**	NA _	NA _	**Neutral** **0.137**	7
A246G	**Neutral** **0.060**	**Neutral** **0.166**	NA _	NA _	**Neutral** **0.118**	8
A246V	**Neutral** **0.282**	**Neutral** **0.123**	NA _	NA _	**Neutral** **0.091**	8
A713G	NA _	**Neutral** **0.078**	NA _	NA _	**Neutral** **0.038**	9
A713V	NA _	**Neutral** **0.265**	NA _	NA _	**Neutral** **0.110**	8
A758G	NA _	*Disease* *0.664*	NA _	NA _	**Neutral** **0.457**	1
A758V	NA _	*Disease* *0.633*	NA _	NA _	**Neutral** **0.462**	1
A820G	**Neutral** **0.373**	*Disease* *0.874*	NA _	NA _	*Disease* *0.694*	4

*For Parkinson’s disease*, SNP analysis was performed on *A. aegypti* LRRK1 protein (XP_021698550.1) as a homolog of *H. sapiens* LRRK2 with 27.47% protein identity*,* using the reference human SNP rs33939927 (R > S,G,C).

1. In rs33939927, Arg1441Ser or Arg1441Gly, or Arg1441Cys in humans matches Arg at position 1218 in *A. aegypti.*

Prediction using the SIFT, PANTHER, SNAP, PhD-SNP, and Meta-SNP tools results from three input suggested mutations. Three mutations showed deleterious or diseased points Table 5. Mutations R1218C, R1218C, and R1218S are thought to be pathogenic in the PD *A. aegypti* model according to Phd-SNP, and Meta-SNP while PANTHER, SIFT and SNAP couldn’t identify the effects of nsSNPs. In spite of the fact that **R1218C** has the highest reliability index.

**Table 5:** Predicted nsSNPs R1218C, R1218C, and R1218S in *A. aegypti* LRRK1 protein. *Italic* for diseased effect and **Bold** for neutral effect.

Mutation	PANTHER	Phd-SNP	SIFT	SNAP	Meta-SNP	RI
R1218C	NA _	*Disease* *0.803*	NA _	NA _	*Disease* *0.673*	3
R1218G	NA _	*Disease* *0.642*	NA _	NA _	*Disease* *0.509*	0
R1218S	NA _	*Disease* *0.691*	NA _	NA _	*Disease* *0.520*	0

*For Huntington’s disease*, SNP analysis was performed on *T. castaneum* VCP-like protein (XP_008192481.1) as a homolog of *H. sapiens* VCP with 43.99% protein identity*,* using the reference human SNPs rs121909330 (R > C,G,S), rs121909334 (R > P,Q), and rs387906789 (R > C,G,S).

1. In rs121909330, Arg155Cys or Arg155Gly, or Arg155Ser in humans matches Arg at positions 268, 282, and 836 in *T. castaneum.*
2. In rs121909334, Arg191Pro or Arg191Gln in human matches 618, 639, 739, 89, 217, 743, 618, 82, 330, 411, and 462 in *T. castaneum.*
3. In rs387906789, Arg159Cys or Arg159Gly, or Arg159Ser in humans matches Arg at positions 710, and 750 in *T. castaneum.*

Prediction using the SIFT, PANTHER, SNAP, PhD-SNP, and Meta-SNP tools results from 37 input-suggested mutations. Fifteen mutations were predicted, thirteen of the 15 mutations showed deleterious or diseased points Table 6. Mutations R268C, R268G, R268S, R282C, R282G, R282S, R836C, R710C, R710G, R710S, R750C, R750G, and R750S are thought to be pathogenic in the HD *T. castaneum* model according to PANTHER, Phd-SNP, SIFT, SNAP and Meta-SNP. In spite of the fact that **R268C, R268G, R282G, R710C, R750C, R750G** have the highest reliability index.

**Table 6:** Predicted nsSNPs R268C, R268G, R268S, R282C, R282G, R282S, R836C, R710C, R710G, R710S, R750C, R750G, and R750S in *T. castaneum* VCPl protein. *Italic* for diseased effect and **Bold** for neutral effect.

Mutation	PANTHER	Phd-SNP	SIFT	SNAP	Meta-SNP	RI
R268C	**Neutral** **0.229**	*Disease* *0.751*	**Neutral** **0.150**	**Neutral** **0.300**	**Neutral** **0.209**	6
R268G	**Neutral** **0.385**	*Disease* *0.639*	**Neutral** **0.210**	**Neutral** **0.500**	**Neutral** **0.229**	5
R268S	**Neutral** **0.246**	*Disease* *0.611*	**Neutral** **0.330**	**Neutral** **0.350**	**Neutral** **0.280**	4
R282C	*Disease* *0.835*	*Disease* *0.807*	*Disease* *0.050*	*Disease* *0.635*	*Disease* *0.696*	4
R282G	*Disease* *0.563*	*Disease* *0.574*	*Disease* *0.000*	*Disease* *0.710*	*Disease* *0.739*	5
R282S	**Neutral** **0.257**	**Neutral** **0.428**	*Disease* *0.000*	*Disease* *0.700*	**Neutral** **0.488**	0
R836C	**Neutral** **0.464**	*Disease* *0.594*	*Disease* *0.000*	*Disease* *0.565*	*Disease* *0.638*	3
R836G	**Neutral** **0.129**	**Neutral** **0.410**	**Neutral** **0.090**	**Neutral** **0.495**	**Neutral** **0.402**	2
R836S	**Neutral** **0.190**	**Neutral** **0.315**	**Neutral** **0.090**	**Neutral** **0.350**	**Neutral** **0.411**	2
R710C	*Disease* *0.892*	*Disease* *0.821*	*Disease* *0.000*	*Disease* *0.605*	*Disease* *0.765*	5
R710G	*Disease* *0.739*	*Disease* *0.678*	*Disease* *0.000*	*Disease* *0.615*	*Disease* *0.676*	4
R710S	*Disease* *0.639*	**Neutral** **0.457**	*Disease* *0.020*	*Disease* *0.520*	*Disease* *0.591*	2
R750C	*Disease* *0.947*	*Disease* *0.912*	*Disease* *0.000*	*Disease* *0.710*	*Disease* *0.803*	6
R750G	*Disease* *0.835*	*Disease* *0.848*	*Disease* *0.000*	*Disease* *0.655*	*Disease* *0.726*	5
R750S	*Disease* *0.820*	*Disease* *0.892*	*Disease* *0.010*	*Disease* *0.625*	*Disease* *0.669*	3

Prediction; Neutral: Neutral variants. Disease: Disease causing variants.

Outputs: Value reported under each prediction.

PANTHER, PhD-SNP, and Meta-SNP: between 0 and 1 (if > 0.5, mutation is predicted disease).

SIFT: Positive Value (If > 0.05, mutation is predicted Neutral). SNAP: Output normalised between 0 and 1 (if > 0.5, mutation is predicted disease).

RI: A Reliability Index between 0 and 10 provides a means of focusing on the most accurate predictions.

## Discussion

Neurodegenerative diseases are devastating diseases which are incurable and mostly result in the death of patients. To accelerate the search for treatments and save money, effort, and time, there is a need to determine the best model that mimics human disease. In turn, this leads to improved human neural health. Pairwise alignment was applied to each protein against humans for all proteins except (GRN and NEP2) against the fruit fly because they showed no alignment against *H. sapiens*. We determined the best insect for studying each protein separately by selecting the highest query coverage with the highest protein identity.

In this study, a total of eight insect models were used to find out which of them is the best to model each of AD, PD and HD.

For Alzheimer’s, the best overall two models according to the average protein identity percentage for the 10 selected proteins were *D. melanogaster* then *A. gambiae. Drosophila melanogaster* is believed to have nearly 75% of human disease-causing genes functional homologs^15,40,41^. The fruitfly showed a high protein identity to human with reasonable query coverage in GRN, COL25A1, MAPT and RAC1. They can express different phenotypes of induced AD^15^. From the 10 proteins, APP was selected as a representative of AD related proteins in human. The analysis of nsSNPs related to APPl protein in the fruit fly showed predicted pathogenic nsSNPs (V94F, V94L, A758G, A758V, and A820G) that could be used for further studies on the induction of familial forms of early-onset Alzheimer's disease and cerebral amyloid angiopathy, and study the factors that increase total Aβ levels^42,43^. *Anopheles gambiae* is known to become an important model organism for the study of insect-parasite interactions and innate immune responses to protozoan parasites^44^. *Anopheles gambiae* shows better protein identity to *H. sapiens* than *D. melanogaster* for DJ-1, VCP and PLA2G6 proteins. Moreover, *A. gambiae* infection with *Toxoplasma gondii* promotes the accumulation of glutamate. Glutamate is a neurotransmitter in the brain that triggers neurodegenerative diseases such as Alzheimer’s disease and Parkinson’s disease in individuals predisposed to such conditions^45^. Thus in turn makes *A. gambiae* a potential model to study the pathology of these AD.

For Parkinson’s, the best two models according to the average protein identity percentage for the 13 selected proteins were *A. aegypti* then *A. mellifera*. *A. aegypti* has an advanced nervous system, with sensory organs used to locate their hosts in their environment^46^. On applying a sublethal dose of spinosyn insecticides to *A. aegypti*. Parkinson's disease-related genes were significantly enriched in spinetoram-exposed mosquitoes compared with controls^47^. Through our studies, it showed a high protein identity to human with reasonable query coverage for PARK6, VPS35, ATP13A2 and PLA2G6. From the 13 proteins, LRRK2 was selected as a representative of PD related proteins in human. The analysis of nsSNPs related to LRRK1 protein in the yellow fever mosquito showed predicted pathogenic nsSNPs (R1218C, R1218C, and R1218S) that could be used for induction of PD through mutations in the catalytic domains that may result in hyperactivation of the kinase domain, and show Lewy Body pathology^48^. *Apis mellifera* is more similar to vertebrates in terms of RNA (Ribonucleic acid) interference, DNA (Deoxyribonucleic Acid) methylation, and circadian rhythm^49^. It showed a high protein identity to human with reasonable query coverage in PARK2, VPS35 and ATP13A2. Honey bees’ ethanol exposure causes changes in their body and wing kinematics^50^. Mechanisms identified in the cellular stress response to ethanol, such as the oxidative stress response, are also involved in Parkinson’s disease^51^. *Apis mellifera* is a key social behavioural model that displays sophisticated cognitive abilities^52^. This makes it possible to analyse the changes occurring in honeybee brains during learning and remembering and increases the opportunity to be used also as a model for AD, along with the ability to identify new genome-based single-nucleotide polymorphisms (SNPs)^14,53^.

For Huntington’s, *T. castaneum* then *B. mori* were the best models according to the average protein identity percentage. *Tribolium castaneum* has more olfactory receptors and detoxification genes than *D. melanogaster* and other insects and may be better adapted to its environment^45^. It shows a higher genetic homology to humans when compared to other invertebrate models, such as *D. melanogaster*^54^. Therefore, *T. castaneum* is one of the most suitable genetic models for post-genomic studies such as proteomics and functional genomics. It showed a high protein identity to human with reasonable query coverage in GRIK2, VPS13A and UBQLN2. From the 10 proteins, VCP was selected as a representative of HD related proteins in human. The analysis of SNPs related to VCPl protein in the Red flour beetle revealed predicted pathogenic nsSNPs (R268C, R268G, R268S, R282C, R282G, R282S, R836C, R710C, R710G, R710S, R750C, R750G, and R750S) that could be used for further studies on the gene role in cell division, the cell apoptosis, repairing damaged DNA, and formation of abnormal proteins build up in muscle, bone and brain cells that lead to induction of HD. These protein aggregations interfere with the normal functions of the brain cells^55,56^. The PINK1 protein from the *T. castaneum* beetle (TcPINK1) exhibits catalytic activity toward ubiquitin, parkin, and generic substrates and provides a basis for further studies on human Parkinson’s disease^57^. *Bombyx mori* shares 58% of diseased human homologs genes, which are related to neurodegenerative diseases such as HD, oxidative stress, and protein degradation-associated genes^58^. *Bombyx mori* has higher identical VPS35, and UCHL1 to *H. sapiens* than *D. melanogaster*. Downregulation of the DJ-1 gene causes p-translucent silkworm as a result of increased oxidative stress response of the body, which leads to oxidative damage to the nerves and tissues^17,18^.

*Galleria mellonella* didn’t represent the best model for any of the three studied NDs, although it has a similar innate immune response to that of mammals, regardless of whether it evolved separately from mammals several thousand years ago^29–31^. Comparative studies of genomes have shown that it has numerous homologues of human genes encoding proteins involved in pathogen recognition or signal transduction^59,60^. According to our study, it showed a high protein identity to human with reasonable query coverage in MAPT, ATP13A2, GIGYF2 and RAC1. In addition, its larvae can cultivate Bacteria such as *Borrelia burgdorferi*^61^, *Enterococcus faecalis*^62^, and *Staphylococcus aureus*^63^, which are believed to play a role in neuroinflammation and may contribute to AD.

*Musca* *domestica* has a strong immune system and has been used as a model to investigate the presence of enhanced detoxification^64^. Applying its larval extract on an AD diseased mouse has therapeutic effects against memory impairment, structural damage, and oxidative stress^65^. According to our study, it showed a high protein identity to human with reasonable query coverage in RAC1, COL25A1, HDAC6, DJ-1, GRIK2, VPS13A, VCP and UBQLN2.

**Figure 5:**
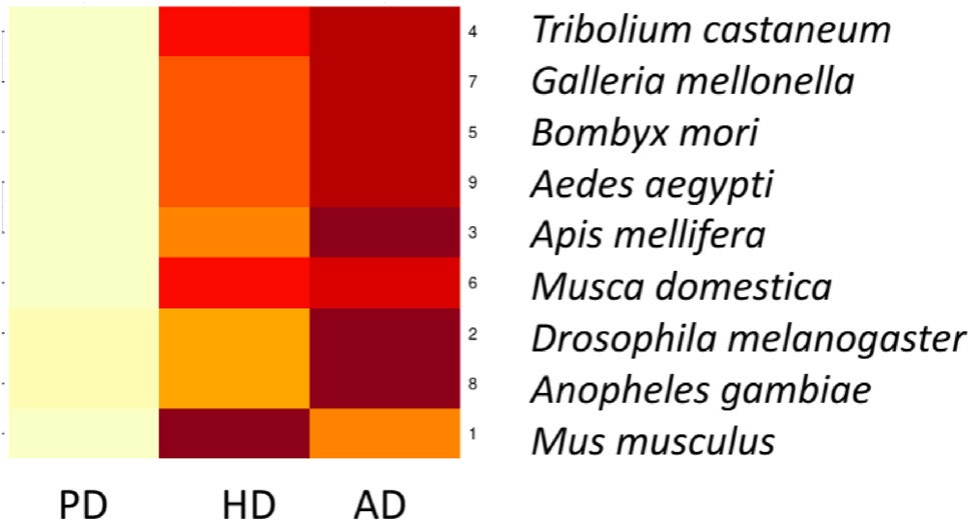
The heatmap shows the percentage of protein identity for AD, PD, and HD between different insect models, Where deep colour refers to high protein identity and light colour refers to low protein identity. The heatmap was generated using RStudio version 2022.12.0 + 353.

**Figure 6:**
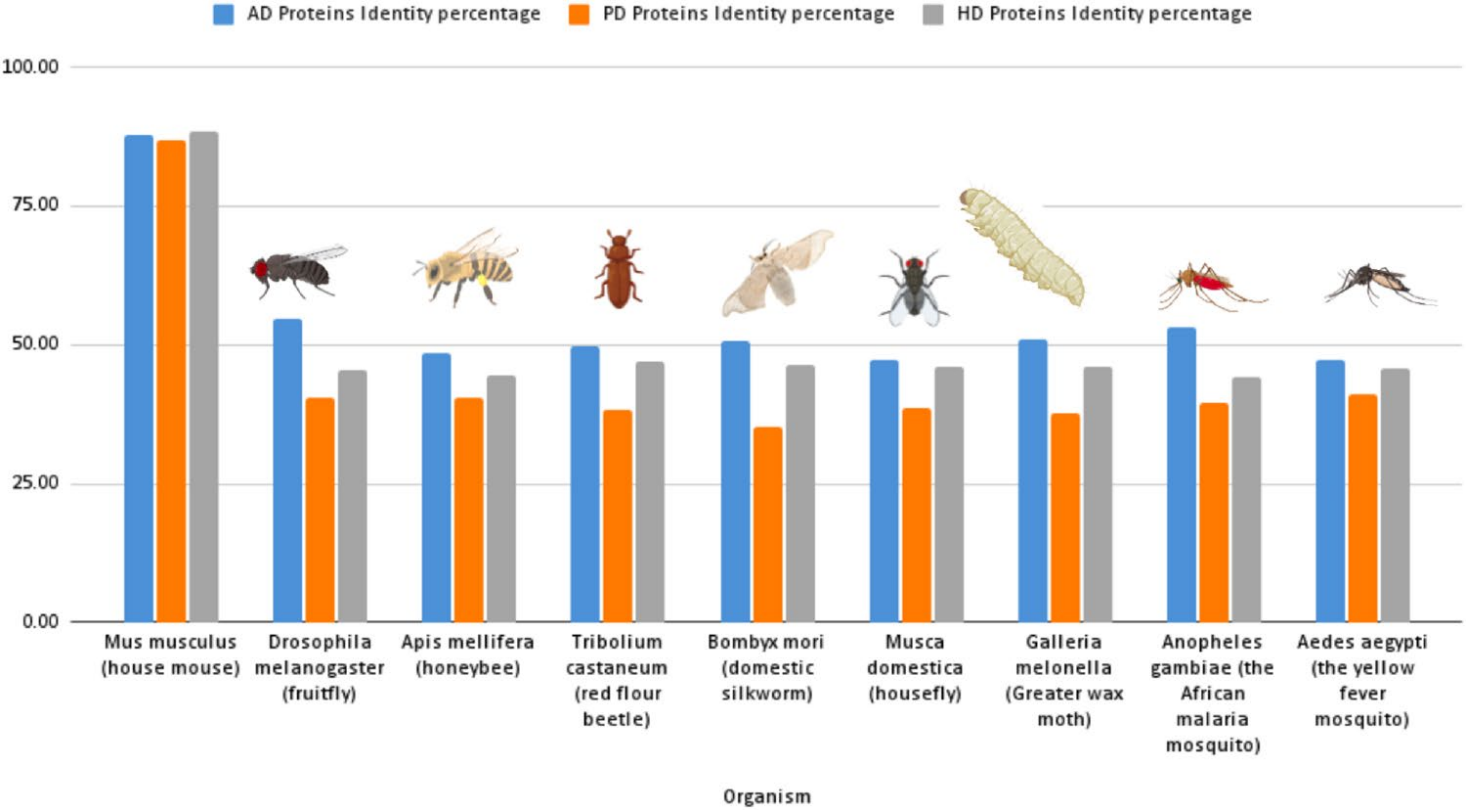
The diagram shows the average protein identity percentage between different selected insect models. The best overall insect models according to protein identity are The Fruit fly for AD, Yellow fever mosquito for PD, and Red flour beetle for HD.

These findings will assist in the selection of the best model for further studies in simulation diseases, deep understanding for mutations and their effects and how to fix them genetically or through improving drug discovery. The average percentage of protein identity between the different insect models and the selected proteins is provided in the supplementary data, as shown in Figs. 5, and 6.

## Conclusion

The increasing prevalence of neurodegenerative diseases such as Alzheimer's, Parkinson’s and Huntington's necessitates improvement in our understanding of these diseases. The research strategy for NDs is two-armed; one of them focuses on finding actual treatments that work on delaying symptoms or preventing disease development, whereas the other depends on searching for tools that can be used to detect the earliest and indirect signs of the disease and this is our point. Thus, it is crucial to simulate the disease, identify the counterparts of human diseased genes, test and apply their findings to easily handled model organisms. Comparative analysis has the potential to improve research and drug development for human diseases.

In this study, a total of 61 SNPs were checked in APPl, LRRK1 and VCPl proteins of *D. melanogaster*, *A. aegypti* and *T. castaneum* respectively by five prediction tools; 21 out of 29 SNPs showed a deleterious effect and 8 of the 21 showed high reliability index. For the 21 deleterious nsSNPs, most of them are located on the functional domains of the proteins.

Although mammalian models are more similar to humans, insects are often preferred because of their shorter lifespan and fewer ethical constraints. Human insect disease models provide new tools for drug discovery to overcome current limitations by using them at different stages as models that show a significant response to many drugs that act on the mammalian central nervous system (CNS) instead of differences in their brains, which allows researchers to find new therapeutic strategies.

In conclusion *A. mellifera, T. castaneum, B. mori, A. aegypti* besides *D. melanogaster* have promising future in the field of medical research and provide valuable insights into common neurodegenerative diseases as AD and PD and rare diseases as HD. This study provides comprehensive information on the available insect models on the protein-level resources and analysis of the predicted functional nsSNPs to improve human neural health by finding the best insect model to study Alzheimer’s disease, Parkinson’s disease, and Huntington’s disease, and to find answers to complex biological questions as the functional impacts of these variants. This will happen by using the findings of the predicted nsSNPs for example to enhance wet-labs experiments and detect the proper position to be knocked down and find out the pathological effects of it and on determining the possible affected genes or proteins on induction of one of the NDs in its proper models.

### Recommendation

To maximise the benefits, we recommend the provision of stock centres of different insect models, mutant and transgenic strains, microarrays, or RNA interference libraries, and working on updating annotations, providing more genome sequencing and assembly of sequenced insects. Additionally, we recommend the development of tools specific to insect model organisms.

### Supplementary Information

Supplementary Information.

## Data Availability

All retrieved data (Human data or models data) are from publicly available databases. All data generated or analysed during this study are included in this published article [and its supplementary information files].
