# Supplementary material for: In silico SNP prediction of selected protein orthologues in insect models for Alzheimer's, Parkinson's, and Huntington’s diseases
Source: Sci Rep. 2023 Nov 3;13:18986. doi: 10.1038/s41598-023-46250-5 (PMC10624829; doi:10.1038/s41598-023-46250-5)
Supplement: Supplementary file 1 — Supplementary Information. [file 41598_2023_46250_MOESM1_ESM.docx]

**Table S1 Table of selected insect models with an overview about their genome updated February 2023.**


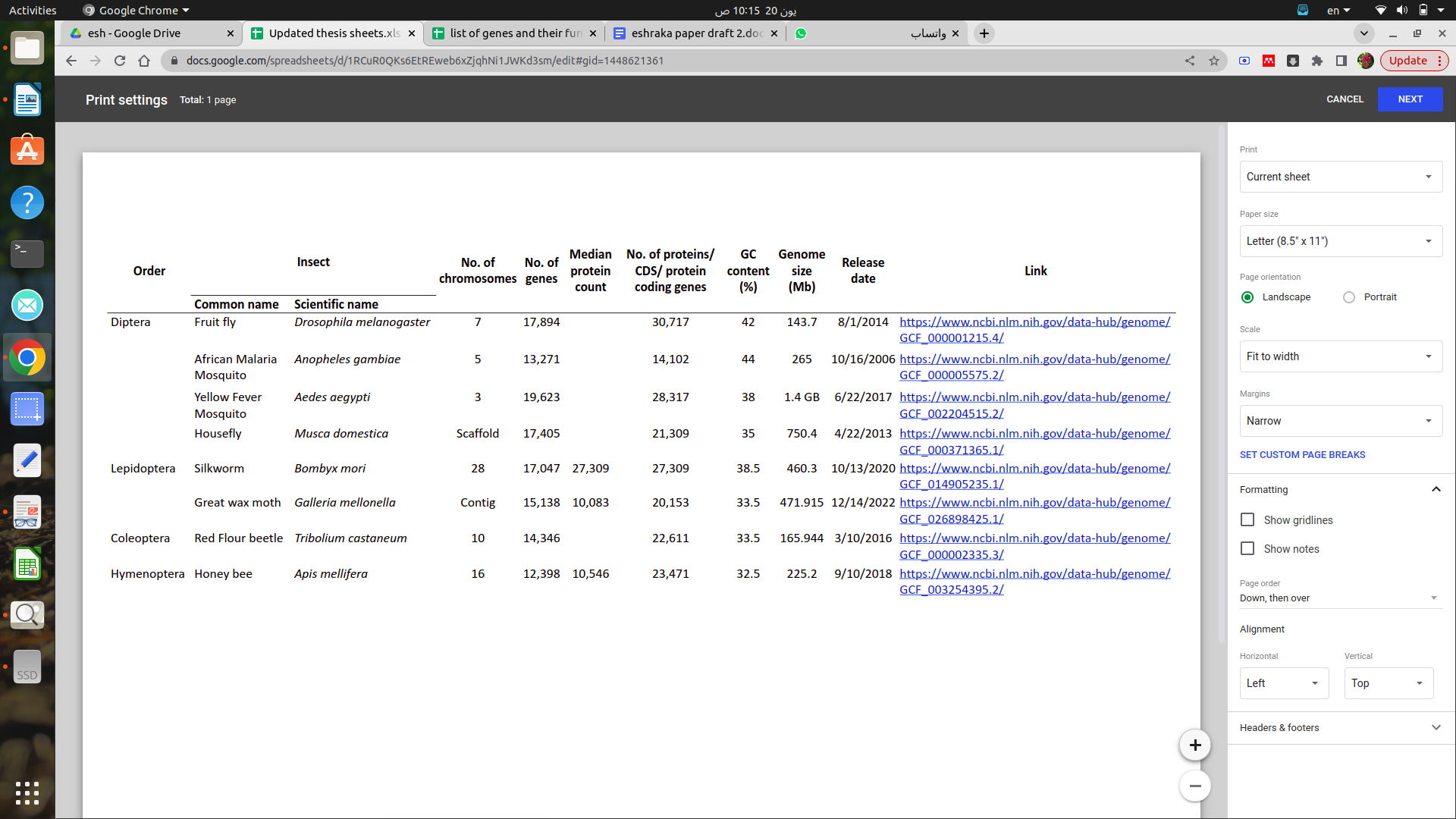


**Table S2 shows the phenotypic characteristics representing AD in *Drosophila melanogaster***

| **Diseases/Gene** | **Phenotypes** |
| --- | --- |
| **Alzheimer’s Disease** |  |
| Amyloid protein | Eye degeneration, Accumulation of amyloid plaques, reduced life span, locomotor defect, and vacuolation of the brain. |
| Presenilin | Pupal lethality, dorsoscutellar bristle duplications, wing notching and wing vein defects. |
| Tau | Eye degeneration, disruption of the microtubular network at presynaptic nerve terminals, axonal degeneration, neuromuscular junctions, morphological defects. |
| **Parkinson’s Disease** |  |
| α-Synuclein | Age-dependent loss of dopaminergic neuron and progressive climbing defect. |
| Parkin and Pink | Dopaminergic neuron loss, age-dependent motor deficits, reduced lifespan, locomotor defects, male sterility and mitochondrial pathology. |
| **Huntington’s disease** |  |
| Triplet repeat expansion | Axonal transport defect, lethality, neurodegeneration, behavioural and electrophysiological defects. |

**Table S3 shows the most contributed genes, their proteins, and protein functions with few mutation effects in AD, PD, and HD.**

| **Diseased Gene** | **Protein** | **Function** | **Mutation effect** | **References** |
| --- | --- | --- | --- | --- |
| Alzheimer's Disease |  |  |  |  |
| APP | Amyloid-beta precursor protein | APP is a type I transmembrane protein whose proteolysis gives rise to β-Amyloid production. APP42-beta may activate mononuclear phagocytes in the brain and elicit inflammatory responses. It has a role in Neuronal development, Memory, Learning, Synaptic formation. | The majority of pathogenic APP mutations cluster near the cleavage sites of the proteases β-secretase and γ-secretase, and generally increase total Aβ levels. Mutations in APP can cause familial forms of early-onset Alzheimer's disease and cerebral amyloid angiopathy. | Müller, U. C., & Zheng, H. (2012). Physiological functions of APP family proteins. Cold Spring Harbor perspectives in medicine, 2(2), a006288.<https://doi.org/10.1101/cshperspect.a006288> Giri, M., Zhang, M., & Lü, Y. (2016). Genes associated with Alzheimer's disease: an overview and current status. Clinical interventions in aging, 11, 665–681.<https://doi.org/10.2147/CIA.S105769> |
| COL25A1 | collagen type XXV alpha 1 chain | This gene encodes a brain-specific membrane associated collagen. | The risk allele carriers had an increased risk for AD. | Forsell, C., Björk, B. F., Lilius, L., Axelman, K., Fabre, S. F., Fratiglioni, L., Winblad, B., & Graff, C. (2010). Genetic association to the amyloid plaque associated protein gene COL25A1 in Alzheimer's disease. Neurobiology of aging, 31(3), 409–415.<https://doi.org/10.1016/j.neurobiolaging.2008.04.009> |
| GRN | Granulin Precursor | Regulate cell growth, neuroinflammation. However, different members of the granulin protein family may act as inhibitors, stimulators, or have dual actions on cell growth. | Loss-of-function mutations in GRN are a cause of familial frontotemporal dementia. | Reho, P., Koga, S., Shah, Z., Chia, R., International LBD Genomics Consortium, American Genome Center, Rademakers, R., Dalgard, C. L., Boeve, B. F., Beach, T. G., Dickson, D. W., Ross, O. A., & Scholz, S. W. (2022). GRN Mutations Are Associated with Lewy Body Dementia. Movement disorders : official journal of the Movement Disorder Society, 37(9), 1943–1948.<https://doi.org/10.1002/mds.29144> |
| HDAC6 | Histone Deacetylase 6 | The protein regulates the acetylation of non-histone proteins involved in intracellular transport, neurotransmitter release, and aggregate formation, and plays a leading role in neuronal health or dysfunction. It also alters chromosome structure and affects transcription factor access to DNA. | Deregulation of HDAC6 has been connected to AD, and PD. | LoPresti P. (2020). HDAC6 in Diseases of Cognition and of Neurons. Cells, 10(1), 12.<https://doi.org/10.3390/cells10010012> |
| MAPT | Microtubule Associated Protein Tau | This gene encodes a membrane protein that forms a receptor signalling complex with the tyrosine kinase binding protein. The encoded protein functions in immune response and may be involved in chronic inflammation by triggering the production of constitutive inflammatory cytokines. | High concentration of Hyperphoshorelated Tau increased risk of AD. Few rare mutations ( p.R47H and p.R62H ) are associated with the AD. | Hoogmartens, J., Cacace, R., & Van Broeckhoven, C. (2021). Insight into the genetic etiology of Alzheimer's disease: A comprehensive review of the role of rare variants. Alzheimer's & dementia (Amsterdam, Netherlands), 13(1), e12155.<https://doi.org/10.1002/dad2.12155> |
| Nep2 | Neprilysin-2 | It degrades amyloid-β (Aβ), serves an important role in clearing Aβ, and plays important roles in pain perception, arterial pressure regulation, phosphate metabolism and homeostasis. | Mutations increase amyloid-β (Aβ). | Huang, J. Y., Hafez, D. M., James, B. D., Bennett, D. A., & Marr, R. A. (2012). Altered NEP2 expression and activity in mild cognitive impairment and Alzheimer's disease. Journal of Alzheimer's disease : JAD, 28(2), 433–441.<https://doi.org/10.3233/JAD-2011-111307> |
| PSN-1 | Presenilin-1 | This protein, an aspartyl protease, is one part (subunit) of a complex called gamma- (γ-) secretase. Presenilin 1 carries out the major function of the complex, which is to cleave other proteins into smaller pieces called peptides. It has a role in γ-Secretase activity, Intracellular signalling, β-Amyloid production, and catalysing the dephosphorylation of nucleoside 5'-monophosphates. | G384A mutation, next to the critical D385, causes higher levels of residues amyloidogenic Aβ42 production | Sreekumar, K. R., Aravind, L., & Koonin, E. V. (2001). Computational analysis of human disease-associated genes and their protein products. Current opinion in genetics & development, 11(3), 247–257.<https://doi.org/10.1016/s0959-437x(00)00186-6> Kelleher, R. J., 3rd, & Shen, J. (2017). Presenilin-1 mutations and Alzheimer's disease. Proceedings of the National Academy of Sciences of the United States of America, 114(4), 629–631.<https://doi.org/10.1073/pnas.1619574114> |
| PSN-2 | Presenilin-2 | Probable catalytic subunit of the gamma-secretase complex, an endoprotease complex that catalyses the intramembrane cleavage of integral membrane proteins such as Notch receptors and APP (amyloid-beta precursor protein), and play a role in intracellular signalling and gene expression or in linking chromatin to the nuclear membrane. | PSEN2 mutation might increase γ-secretase activity. | Cai, Y., An, S. S., & Kim, S. (2015). Mutations in presenilin 2 and its implications in Alzheimer's disease and other dementia-associated disorders. Clinical interventions in aging, 10, 1163–1172.<https://doi.org/10.2147/CIA.S85808> |
| RAC1 | Rac Family Small GTPase 1 | In its active state, binds to a variety of effector proteins to regulate cellular responses such as secretory processes, phagocytosis of apoptotic cells, epithelial cell polarisation, neurons adhesion, migration and differentiation, and growth-factor induced formation of membrane ruffles | Rac1 activity increased 2.5-fold in AD patients compared to that in healthy controls | Reijnders, M. R. F., Ansor, N. M., Kousi, M., Yue, W. W., Tan, P. L., Clarkson, K., Clayton-Smith, J., Corning, K., Jones, J. R., Lam, W. W. K., Mancini, G. M. S., Marcelis, C., Mohammed, S., Pfundt, R., Roifman, M., Cohn, R., Chitayat, D., Deciphering Developmental Disorders Study, Millard, T. H., Katsanis, N., … Banka, S. (2017). RAC1 Missense Mutations in Developmental Disorders with Diverse Phenotypes. American journal of human genetics, 101(3), 466–477.<https://doi.org/10.1016/j.ajhg.2017.08.007> Wu, W., Du, S., Shi, W., Liu, Y., Hu, Y., Xie, Z., Yao, X., Liu, Z., Ma, W., Xu, L., Ma, C., & Zhong, Y. (2019). Inhibition of Rac1-dependent forgetting alleviates memory deficits in animal models of Alzheimer's disease. Protein & cell, 10(10), 745–759.<https://doi.org/10.1007/s13238-019-0641-0> |
| SORL1 | Sortilin-related receptor | Cholesterol metabolism, Processing of APP, as an intracellular sorting receptor as APP is being trafficked between the secretory pathway, Endocytosis, Amyloid β clearance, and Receptor for APOE. | SNPs in SORLA to increase risk of AD, Reduced brain levels of SORLA are thought to alter the transport and processing of APP to increase generation of Aβ peptides. | Pallesen, L. T., & Vaegter, C. B. (2012). Sortilin and SorLA regulate neuronal sorting of trophic and dementia-linked proteins. Molecular neurobiology, 45(2), 379–387.<https://doi.org/10.1007/s12035-012-8236-2> Alvarez-Mora, M. I., Blanco-Palmero, V. A., Quesada-Espinosa, J. F., Arteche-Lopez, A. R., Llamas-Velasco, S., Palma Milla, C., Lezana Rosales, J. M., Gomez-Manjon, I., Hernandez-Lain, A., Jimenez Almonacid, J., Gil-Fournier, B., Ramiro-León, S., González-Sánchez, M., Herrero-San Martín, A. O., Pérez-Martínez, D. A., Gómez-Tortosa, E., Carro, E., Bartolomé, F., Gomez-Rodriguez, M. J., Sanchez-Calvin, M. T., … Moreno-Garcia, M. (2022). Heterozygous and Homozygous Variants in SORL1 Gene in Alzheimer's Disease Patients: Clinical, Neuroimaging and Neuropathological Findings. International journal of molecular sciences, 23(8), 4230.<https://doi.org/10.3390/ijms23084230> (Yin et al. 2015). |
| Parkinson's Disease |  |  |  |  |
| PRKN / PARK2 | Parkin | E3 ubiquitin-protein ligase ubiquitinates outer mitochondrial membrane proteins. | Mutations in the ubiquitin domain affect binding to target proteins; mutations are the most common cause of early-onset PD (EOPD). | Jia, F., Fellner, A., & Kumar, K. R. (2022). Monogenic Parkinson’s Disease: Genotype, Phenotype, Pathophysiology, and Genetic Testing. Genes, 13(3), 471.<https://doi.org/10.3390/genes13030471> |
| Pink1 / PARK6 | PTEN induced putative kinase 1 | It phosphorylates parkin and maintains its mitochondrial stabilisation and translocation, mediating parkin  activation. | Mutations disturb PINK1 which is the selective degradation of mitochondria. | Grünewald, A., Kumar, K. R., & Sue, C. M. (2019). New insights into the complex role of mitochondria in Parkinson's disease. Progress in neurobiology, 177, 73–93.<https://doi.org/10.1016/j.pneurobio.2018.09.003> |
| PARK7 / DJ-1 | Parkinsonism associated deglycase | DJ-1 has a Redox function through scavenging reactive oxygen species, regulation of transcription and signal transduction pathways, and acting as a  molecular chaperone and enzyme, to protect dopaminergic neurons against neurodegeneration. | Mutations (downregulation) affect the survival of cells in oxidative environments. | Dolgacheva, L. P., Berezhnov, A. V., Fedotova, E. I., Zinchenko, V. P., & Abramov, A. Y. (2019). Role of DJ-1 in the mechanism of pathogenesis of Parkinson's disease. Journal of bioenergetics and biomembranes, 51(3), 175–188.<https://doi.org/10.1007/s10863-019-09798-4> Buneeva, O. A., & Medvedev, A. E. (2021). DJ-1 Protein and Its Role in the Development of Parkinson's Disease: Studies on Experimental Models. Biochemistry. Biokhimiia, 86(6), 627–640.<https://doi.org/10.1134/S000629792106002X> |
| GAK | cyclin G associated kinase | Cell cycle regulator. It modifies α-synuclein expression levels. | GAK rs1564282 C/T polymorphism is associated with increased susceptibility to PD. | Pankratz, N., Wilk, J. B., Latourelle, J. C., DeStefano, A. L., Halter, C., Pugh, E. W., ... & PSG—PROGENI and GenePD Investigators, Coordinators and Molecular Genetic Laboratories. (2009). Genomewide association study for susceptibility genes contributing to familial Parkinson disease. Human genetics, 124, 593-605. Ma, Z. G., He, F., & Xu, J. (2015). Quantitative assessment of the association between GAK rs1564282 C/T polymorphism and the risk of Parkinson's disease. Journal of clinical neuroscience : official journal of the Neurosurgical Society of Australasia, 22(7), 1077–1080.<https://doi.org/10.1016/j.jocn.2014.12.014> |
| PARK17 / VPS35 | vacuolar protein sorting  35 | It is involved in the neuronal vesicular recycling from endosomes to the trans-Golgi network. | Compromise the intracellular localization and stability of cell organelles. | Mohan, M., & Mellick, G. D. (2017). Role of the VPS35 D620N mutation in Parkinson's disease. Parkinsonism & related disorders, 36, 10–18.<https://doi.org/10.1016/j.parkreldis.2016.12.001> Williams, E. T., Chen, X., & Moore, D. J. (2017). VPS35, the Retromer Complex and Parkinson's Disease. Journal of Parkinson's disease, 7(2), 219–233.<https://doi.org/10.3233/JPD-161020> |
| PARK5 / UCHL1 | ubiquitin C-terminal hydrolase L1 | This gene is specifically expressed in the neurons and in cells of the diffuse neuroendocrine system, and have a role in Ubiquitin-proteasome | Mutations in this gene may be associated with Parkinson disease. | Mi, Z., & Graham, S. H. (2023). Role of UCHL1 in the pathogenesis of neurodegenerative diseases and brain injury. Ageing research reviews, 86, 101856.<https://doi.org/10.1016/j.arr.2023.101856> |
| PARK18 / EIF4G1 | eukaryotic translation initiation factor 4 gamma 1 | Associated with PD but unlikely to be pathogenic and have a role in translation of mRNA | Mutations in this gene may be associated with Parkinson disease. | Saini, P., Rudakou, U., Yu, E., Ruskey, J. A., Asayesh, F., Laurent, S. B., Spiegelman, D., Fahn, S., Waters, C., Monchi, O., Dauvilliers, Y., Dupré, N., Greenbaum, L., Hassin-Baer, S., Espay, A. J., Rouleau, G. A., Alcalay, R. N., Fon, E. A., Postuma, R. B., & Gan-Or, Z. (2021). Association study of DNAJC13, UCHL1, HTRA2, GIGYF2, and EIF4G1 with Parkinson's disease. Neurobiology of aging, 100, 119.e7–119.e13.<https://doi.org/10.1016/j.neurobiolaging.2020.10.019> |
| PARK9 / ATP13A2 | ATPase cation transporting 13A2 | It transports inorganic cations as well as other substrates. | Mutations in this gene are associated with early onset forms of Parkinson disease. | Park, J. S., Blair, N. F., & Sue, C. M. (2015). The role of ATP13A2 in Parkinson's disease: Clinical phenotypes and molecular mechanisms. Movement disorders : official journal of the Movement Disorder Society, 30(6), 770–779.<https://doi.org/10.1002/mds.26243>, Fujii, T., Nagamori, S., Wiriyasermkul, P., Zheng, S., Yago, A., Shimizu, T., Tabuchi, Y., Okumura, T., Fujii, T., Takeshima, H., & Sakai, H. (2023). Parkinson's disease-associated ATP13A2/PARK9 functions as a lysosomal H+,K+-ATPase. Nature communications, 14(1), 2174.<https://doi.org/10.1038/s41467-023-37815-z> |
| PARK11 / GIGYF2 | GRB10 interacting GYF protein 2 | The encoded protein involved in the regulation of tyrosine kinase receptor signalling. | Mutations in this gene are associated with late onset Parkinson disease. | Zhang, Y., Sun, Q. Y., Yu, R. H., Guo, J. F., Tang, B. S., & Yan, X. X. (2015). The contribution of GIGYF2 to Parkinson's disease: a meta-analysis. Neurological sciences : official journal of the Italian Neurological Society and of the Italian Society of Clinical Neurophysiology, 36(11), 2073–2079.<https://doi.org/10.1007/s10072-015-2316-9> |
| PARK13 / HTRA2 | HtrA serine peptidase 2 | It provides a functional link between proteolytic stress and mitochondrial function. | Mutations in this gene may be associated with Parkinson disease. | Strauss, K. M., Martins, L. M., Plun-Favreau, H., Marx, F. P., Kautzmann, S., Berg, D., ... & Krüger, R. (2005). Loss of function mutations in the gene encoding Omi/HtrA2 in Parkinson's disease. Human molecular genetics, 14(15), 2099-2111. |
| PARK14 / PLA2G6 | phospholipase A2 group VI | It catalyses the release of fatty acids from phospholipids, and has a role in cell membrane homeostasis, mitochondrial  function, fatty acid oxidation, and calcium signalling. | Mutations originally described in neurodegeneration with brain iron accumulation but can also cause autosomal recessive PD, and usually related to younger onset Parkinsonism | Magrinelli, F., Mehta, S., Di Lazzaro, G., Latorre, A., Edwards, M. J., Balint, B., Basu, P., Kobylecki, C., Groppa, S., Hegde, A., Mulroy, E., Estevez-Fraga, C., Arora, A., Kumar, H., Schneider, S. A., Lewis, P. A., Jaunmuktane, Z., Revesz, T., Gandhi, S., Wood, N. W., … Bhatia, K. P. (2022). Dissecting the Phenotype and Genotype of PLA2G6-Related Parkinsonism. Movement disorders : official journal of the Movement Disorder Society, 37(1), 148–161.<https://doi.org/10.1002/mds.28807> |
| PARK15 / FBXO7 | F-box protein 7 | It encodes an adaptor protein involved in substrate degradation and in mitochondrial maintenance interacting with PINK1 and Parkin. The F-box proteins constitute one of the four subunits of the ubiquitin protein ligase complex, which function in phosphorylation-dependent ubiquitination. | Biallelic missense, splice site, and nonsense mutations contribute to juvenile PD. The loss of FBXO7 expression led to a significant inhibition of parkin recruitment to depolarized mitochondria. | Keller Sarmiento, I. J., Afshari, M., Kinsley, L., Silani, V., Akhtar, R. S., Simuni, T., Lubbe, S. J., Krainc, D., & Mencacci, N. E. (2022). Novel bi-allelic FBXO7 variants in a family with early-onset typical Parkinson's disease. Parkinsonism & related disorders, 104, 88–90.<https://doi.org/10.1016/j.parkreldis.2022.10.014> |
| LRRK2 / PARK8 | leucine rich repeat kinase 2 | The protein is present largely in the cytoplasm but also associates with the mitochondrial outer membrane and has a Lysosomal function. | Most patients with the p.G2019S mutation in the catalytic domains may result in hyperactivation of the kinase domain, shows Lewy Body pathology. It has a prevalence of 1% in the PD population with a high prevalence in North African Berber Arab (39%). | Jia, F., Fellner, A., & Kumar, K. R. (2022). Monogenic Parkinson’s disease: genotype, phenotype, pathophysiology, and genetic testing. Genes, 13(3), 471.<https://doi.org/10.3390/genes13030471> |
| Huntington's Disease |  |  |  |  |
| HTT | Huntingtin | It is widely expressed with high expression in the brain. It is required for normal development, mediated trafficking of vesicles and organelles along axons, and Antiapoptotic activity  with neuroprotective functions during numerous proapoptotic challenges. | The genetic defect leading to Huntington's disease may not necessarily eliminate transcription, but may confer a new property on the mRNA or alter the function of the protein. | Schulte, J., & Littleton, J. T. (2011). The biological function of the Huntingtin protein and its relevance to Huntington's Disease pathology. Current trends in neurology, 5, 65–78. |
| DMBK/ DM | Dystrophia myotonica protein kinase | It enables ATP and metal ion binding, myosin phosphatase regulator activity, and protein serine/threonine kinase activity. Also it regulates the production and function of important structures inside muscle cells by interacting with other proteins. | Unstable trinucleotide (CTG) expansion at the DMPK gene locus causes cognitive dysfunctions, and myotonic dystrophy. | Breton, É., Légaré, C., Overend, G., Guay, S. P., Monckton, D., Mathieu, J., Gagnon, C., Richer, L., Gallais, B., & Bouchard, L. (2020). DNA methylation at the DMPK gene locus is associated with cognitive functions in myotonic dystrophy type 1. Epigenomics, 12(23), 2051–2064.<https://doi.org/10.2217/epi-2020-0328> |
| GRIK2 | Glutamate ionotropic receptor kainate type subunit 2 | It functions as ligand-activated ion channels activated by the neurotransmitter glutamate; and plays a vital role in neural development and central nervous system function | Bi-allelic loss of function of the KAR-encoding gene GRIK2 causes a neurodevelopmental disorder | Stolz, J. R., Foote, K. M., Veenstra-Knol, H. E., Pfundt, R., Ten Broeke, S. W., de Leeuw, N., Roht, L., Pajusalu, S., Part, R., Rebane, I., Õunap, K., Stark, Z., Kirk, E. P., Lawson, J. A., Lunke, S., Christodoulou, J., Louie, R. J., Rogers, R. C., Davis, J. M., Innes, A. M., … Swanson, G. T. (2021). Clustered mutations in the GRIK2 kainate receptor subunit gene underlie diverse neurodevelopmental disorders. American journal of human genetics, 108(9), 1692–1709.<https://doi.org/10.1016/j.ajhg.2021.07.007>, Guzmán, Y. F., Ramsey, K., Stolz, J. R., Craig, D. W., Huentelman, M. J., Narayanan, V., & Swanson, G. T. (2017). A gain-of-function mutation in the GRIK2 gene causes neurodevelopmental deficits. Neurology. Genetics, 3(1), e129.<https://doi.org/10.1212/NXG.0000000000000129> |
| VCP | Valosin containing protein | As part of the ubiquitin-proteasome system, it degrades unneeded proteins within cells. It has a role in cell division, prevents the cell apoptosis, and repairs damaged DNA. | Mutated gene ( p97/VCP ) causes excess and abnormal proteins build up in muscle, bone, and brain cells. These protein aggregations interfere with the normal functions of these cells. | Ju, J. S., Miller, S. E., Hanson, P. I., & Weihl, C. C. (2008). Impaired protein aggregate handling and clearance underlie the pathogenesis of p97/VCP-associated disease. The Journal of biological chemistry, 283(44), 30289–30299.<https://doi.org/10.1074/jbc.M805517200>, Meyer, H., & Weihl, C. C. (2014). The VCP/p97 system at a glance: connecting cellular function to disease pathogenesis. Journal of cell science, 127(Pt 18), 3877–3883.<https://doi.org/10.1242/jcs.093831> |
| VPS13A | Vacuolar protein sorting 13 homolog A | It may control steps in the cycling of proteins through the trans-Golgi network to endosomes, lysosomes and the plasma membrane as bridges for lipids to move directionally and in bulk between organellar membranes. | Mutations in this gene cause the autosomal recessive disorder, chorea-acanthocytosis. | Adlakha, J., Hong, Z., Li, P., & Reinisch, K. M. (2022). Structural and biochemical insights into lipid transport by VPS13 proteins. The Journal of cell biology, 221(5), e202202030.<https://doi.org/10.1083/jcb.202202030>, Garrido-Fernández, A., Santos-Lasaosa, S., Bellosta-Diago, E., & Sáchez-Valiente, S. (2020). New pathogenic mutation of chorea-acanthocytosis. Nueva mutación patogénica de corea-acantocitosis. Neurologia, 35(2), 73–74.<https://doi.org/10.1016/j.nrl.2018.03.012> |
| SCA1/ATXN1 | Spinocerebellar ataxia 1 | It involved in regulating various aspects of producing proteins, including the first stage of protein production (transcription) and processing RNA | Alleles with 40 or more CAG repeats in HTT are fully penetrant and cause HD | Zoghbi, H. Y., & Orr, H. T. (2009). Pathogenic mechanisms of a polyglutamine-mediated neurodegenerative disease, spinocerebellar ataxia type 1. The Journal of biological chemistry, 284(12), 7425–7429.<https://doi.org/10.1074/jbc.R800041200>, Akçimen, F., Ross, J. P., Liao, C., Spiegelman, D., Dion, P. A., & Rouleau, G. A. (2021). Expanded CAG Repeats in ATXN1, ATXN2, ATXN3, and HTT in the 1000 Genomes Project. Movement disorders : official journal of the Movement Disorder Society, 36(2), 514–518.<https://doi.org/10.1002/mds.28341> |
| SCA2 / ALS | Spinocerebellar ataxia 2 | It plays a role in the translation of genetic information to produce proteins. | Alleles with 40 or more CAG repeats in HTT are fully penetrant and cause HD | Castilhos, R. M., Souza, A. F., Furtado, G. V., Gheno, T. C., Silva, A. L., Vargas, F. R., Lima, M. A., Barsottini, O., Pedroso, J. L., Godeiro, C., Jr, Salarini, D., Pereira, E. T., Lin, K., Toralles, M. B., Saute, J. A., Rieder, C. R., Quintas, M., Sequeiros, J., Alonso, I., Saraiva-Pereira, M. L., … Jardim, L. B. (2014). Huntington disease and Huntington disease-like in a case series from Brazil. Clinical genetics, 86(4), 373–377.<https://doi.org/10.1111/cge.12283>, Akçimen, F., Ross, J. P., Liao, C., Spiegelman, D., Dion, P. A., & Rouleau, G. A. (2021). Expanded CAG Repeats in ATXN1, ATXN2, ATXN3, and HTT in the 1000 Genomes Project. Movement disorders : official journal of the Movement Disorder Society, 36(2), 514–518.<https://doi.org/10.1002/mds.28342> |
| SCA3 / MJD / ATXN3 | Spinocerebellar ataxia 3 | It is involved in a mechanism called the ubiquitin-proteasome system that destroys and gets rid of excess or damaged proteins. | Alleles with 40 or more CAG repeats in HTT are fully penetrant and cause HD | McLoughlin, H. S., Moore, L. R., & Paulson, H. L. (2020). Pathogenesis of SCA3 and implications for other polyglutamine diseases. Neurobiology of disease, 134, 104635.<https://doi.org/10.1016/j.nbd.2019.104635>, Akçimen, F., Ross, J. P., Liao, C., Spiegelman, D., Dion, P. A., & Rouleau, G. A. (2021). Expanded CAG Repeats in ATXN1, ATXN2, ATXN3, and HTT in the 1000 Genomes Project. Movement disorders : official journal of the Movement Disorder Society, 36(2), 514–518.<https://doi.org/10.1002/mds.28343> |
| UBQLN2 | Ubiquilin 2 | It encodes an ubiquitin-like protein, and physically associates with both proteasomes and ubiquitin ligases. | Mutations included in Huntington's disease. | Zeng, L., Wang, B., Merillat, S. A., Minakawa, E. N., Perkins, M. D., Ramani, B., Tallaksen-Greene, S. J., Costa, M. D. C., Albin, R. L., & Paulson, H. L. (2015). Differential recruitment of UBQLN2 to nuclear inclusions in the polyglutamine diseases HD and SCA3. Neurobiology of disease, 82, 281–288.<https://doi.org/10.1016/j.nbd.2015.06.017> |
| CACNA1A | Calcium voltage-gated channel subunit alpha1 A | Voltage-dependent calcium channels mediate the entry of calcium ions into excitable cells, and are also involved in a variety of calcium-dependent processes, including muscle contraction, hormone or neurotransmitter release, and gene expression | Mutations included in Huntington's disease. | Keo, A., Aziz, N. A., Dzyubachyk, O., van der Grond, J., van Roon-Mom, W. M. C., Lelieveldt, B. P. F., Reinders, M. J. T., & Mahfouz, A. (2017). Co-expression Patterns between ATN1 and ATXN2 Coincide with Brain Regions Affected in Huntington's Disease. Frontiers in molecular neuroscience, 10, 399.<https://doi.org/10.3389/fnmol.2017.00399>, Kordasiewicz, H. B., & Gomez, C. M. (2007). Molecular pathogenesis of spinocerebellar ataxia type 6. Neurotherapeutics : the journal of the American Society for Experimental NeuroTherapeutics, 4(2), 285–294.<https://doi.org/10.1016/j.nurt.2007.01.003> |

**Tables S4, S5, S6 and S7 show the protein identity percentages between human and other selected insect models in PD, AD, HD respectively, and the average of protein identity of each insect model against humans.**

**
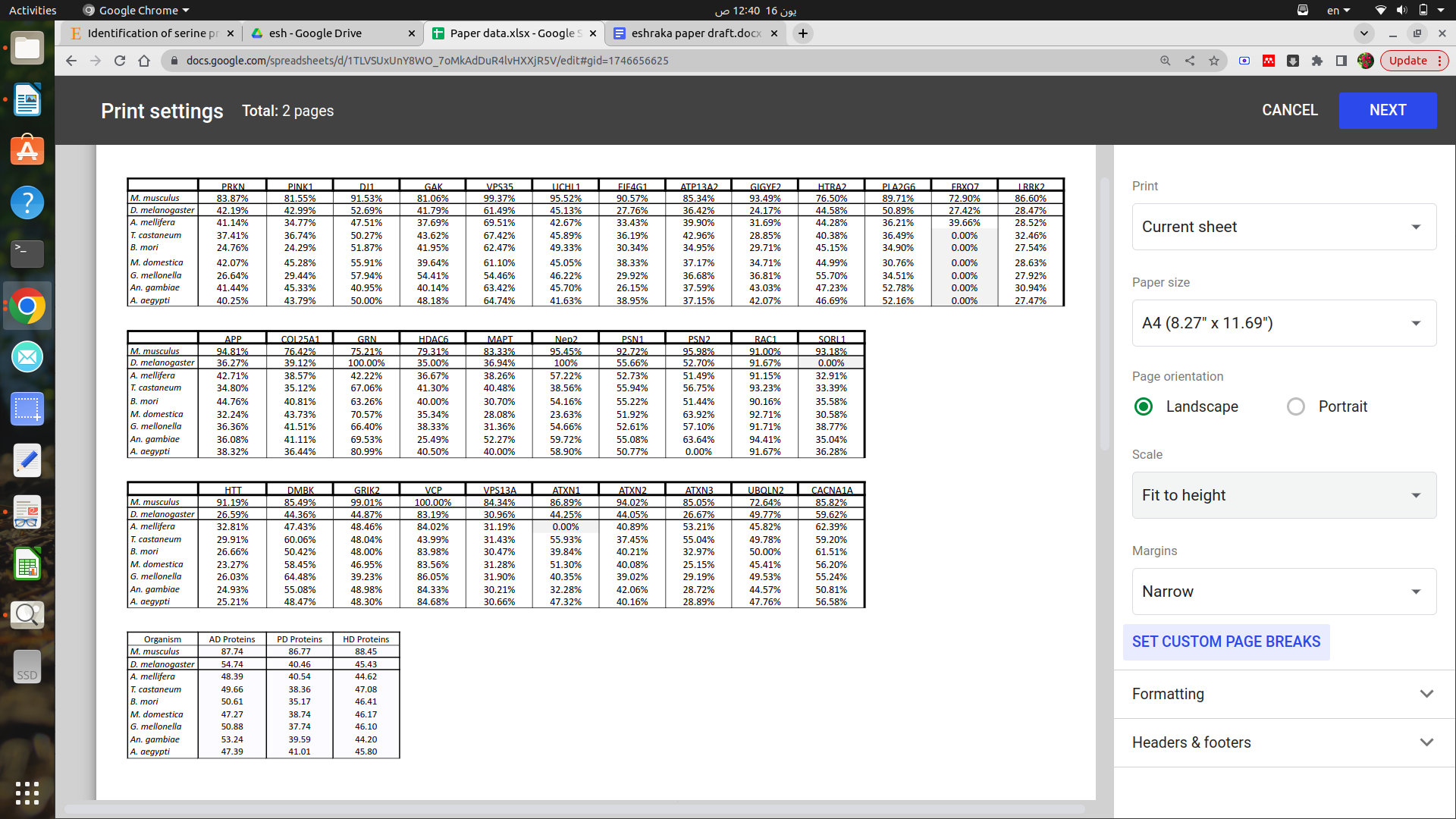
**


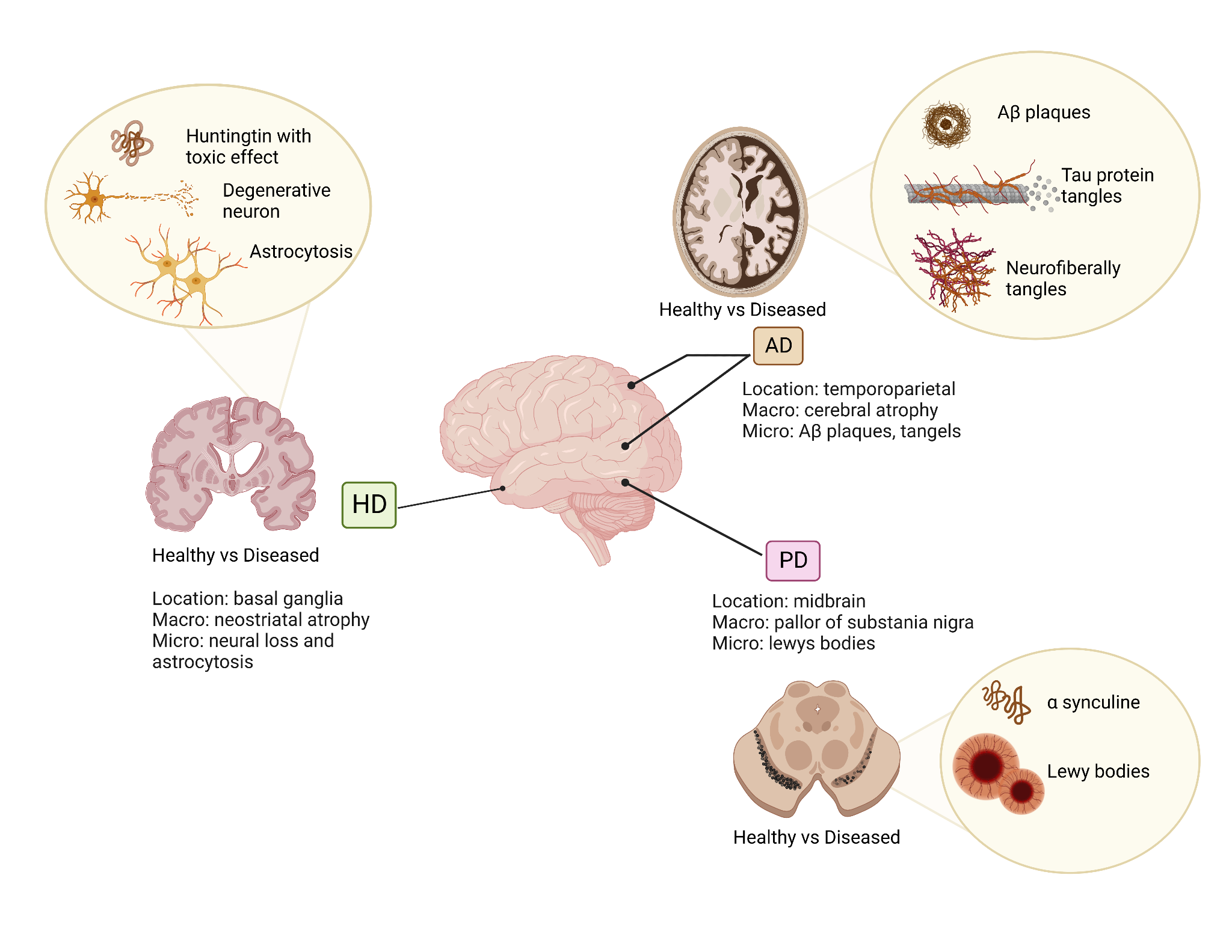


**Figure S1 Diagrammatic representation of the anatomical location and the characteristic hallmarks for Alzhaimer’s, Parkinson’s, and Huntington’s diseases in the human brain. The brain's cross-sections represent the left healthy side against the right diseased side. Created with BioRender.com**


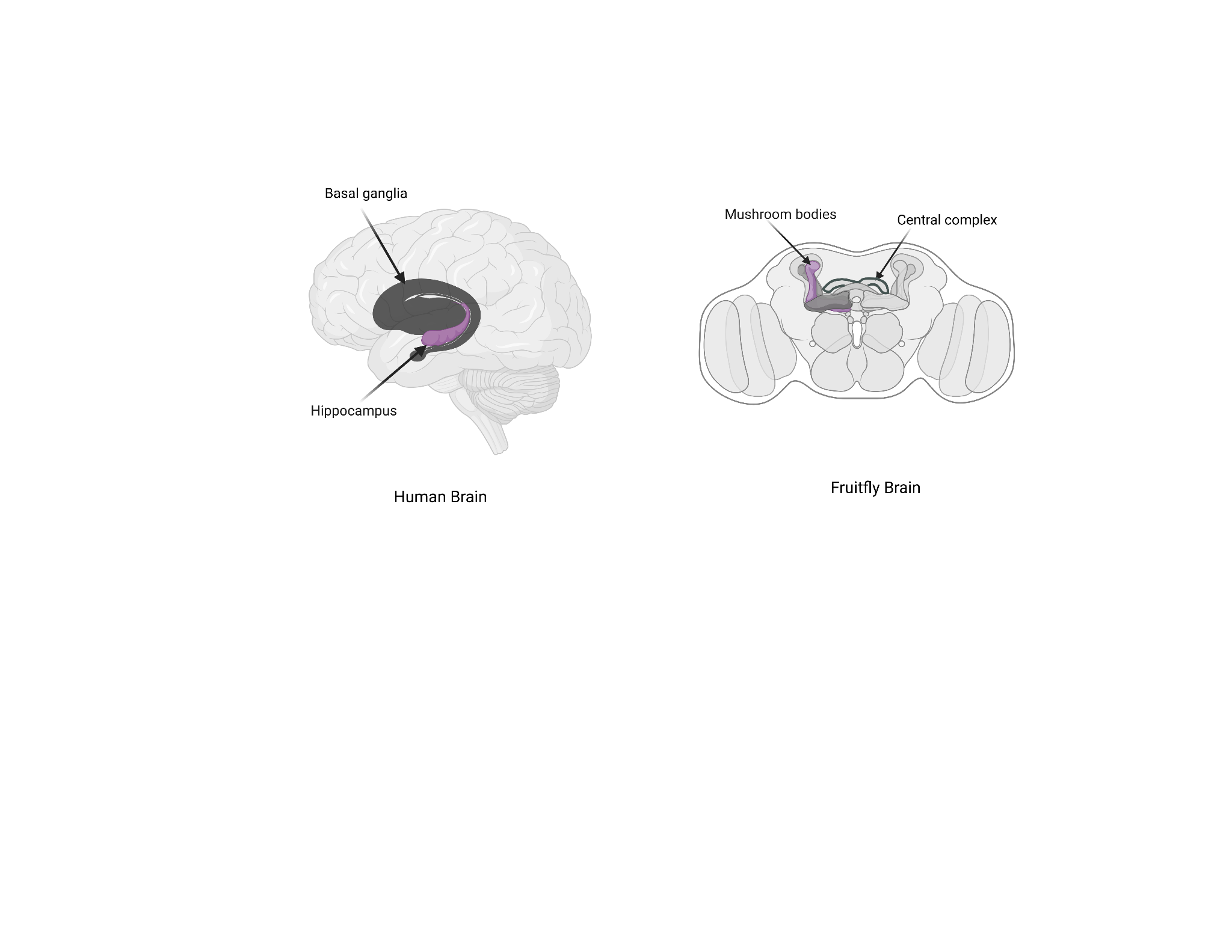


**Figure S2 Structural and functional similarities between humans and fruit fly brains. The central complex in fruit flies parallels the basal ganglia in humans in dark grey, the mushroom bodies in fruit flies parallels hippocampus in humans in pink colour. Created with BioRender.com**

**
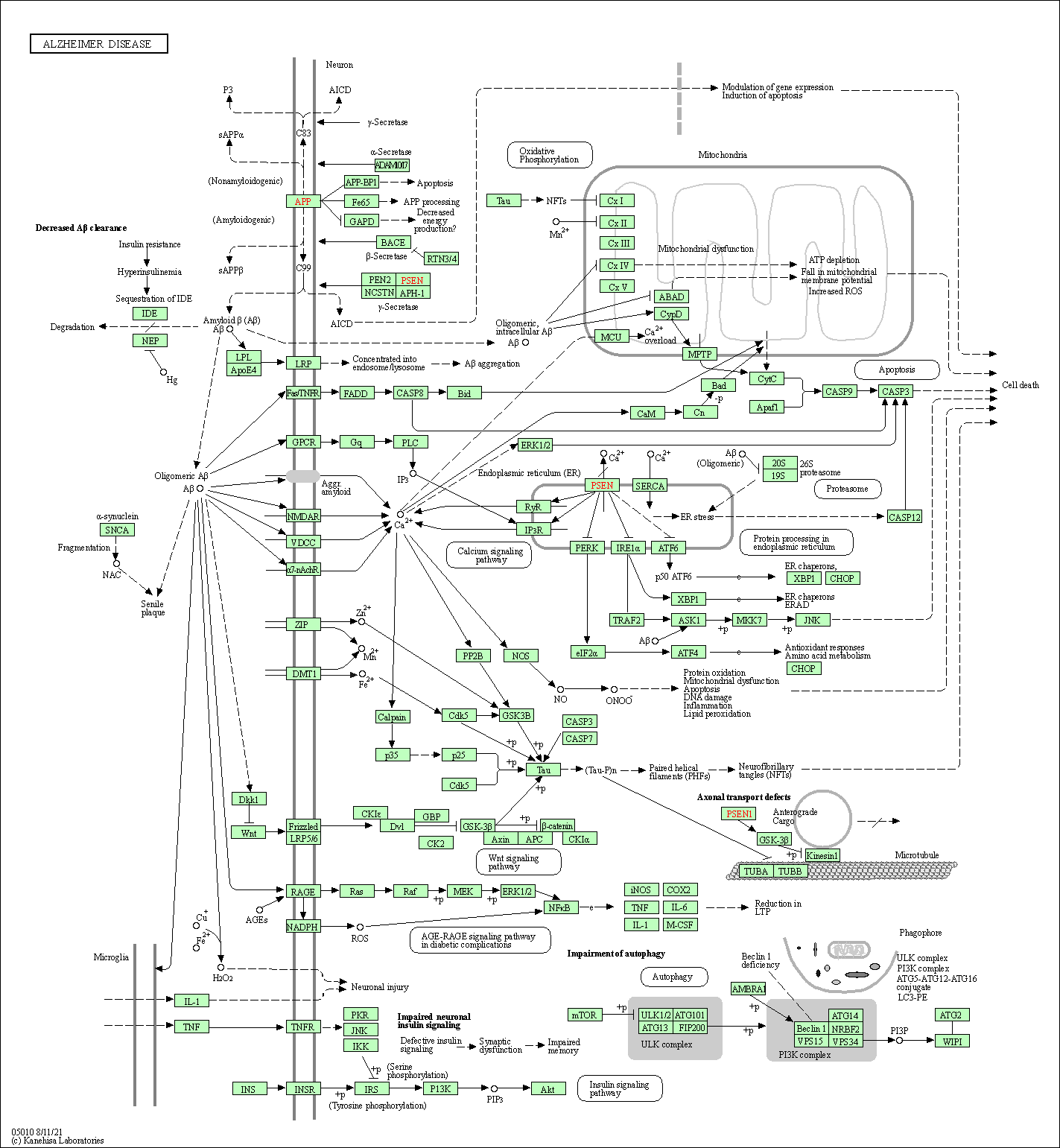
**

**Figure S3 KEGG’S pathways shows contributed genes in pathways related to Alzheimer’s disease**

**
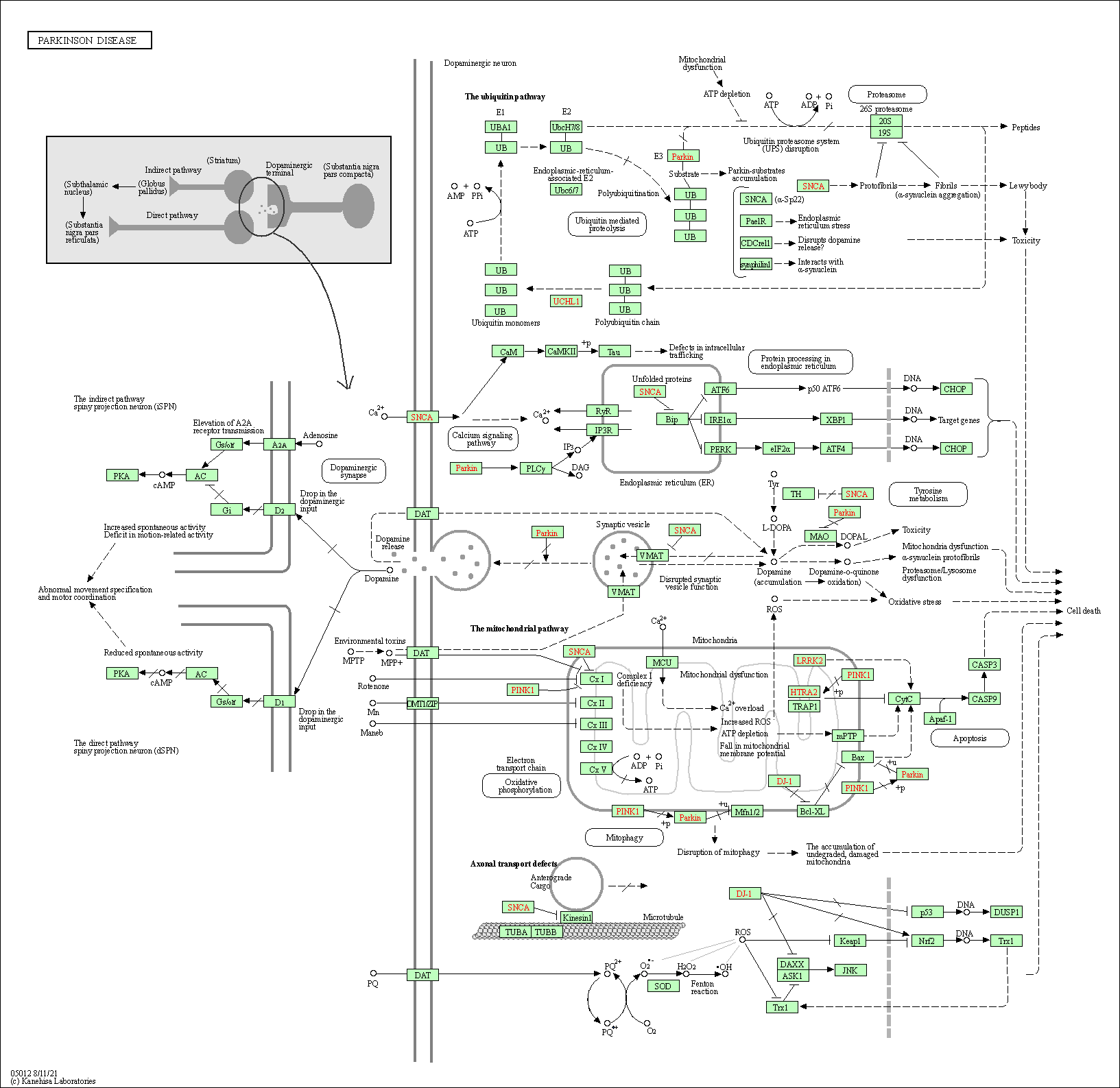
**

**Figure S4 KEGG’S pathways shows contributed genes in pathways related to Parkinson’s disease**

**
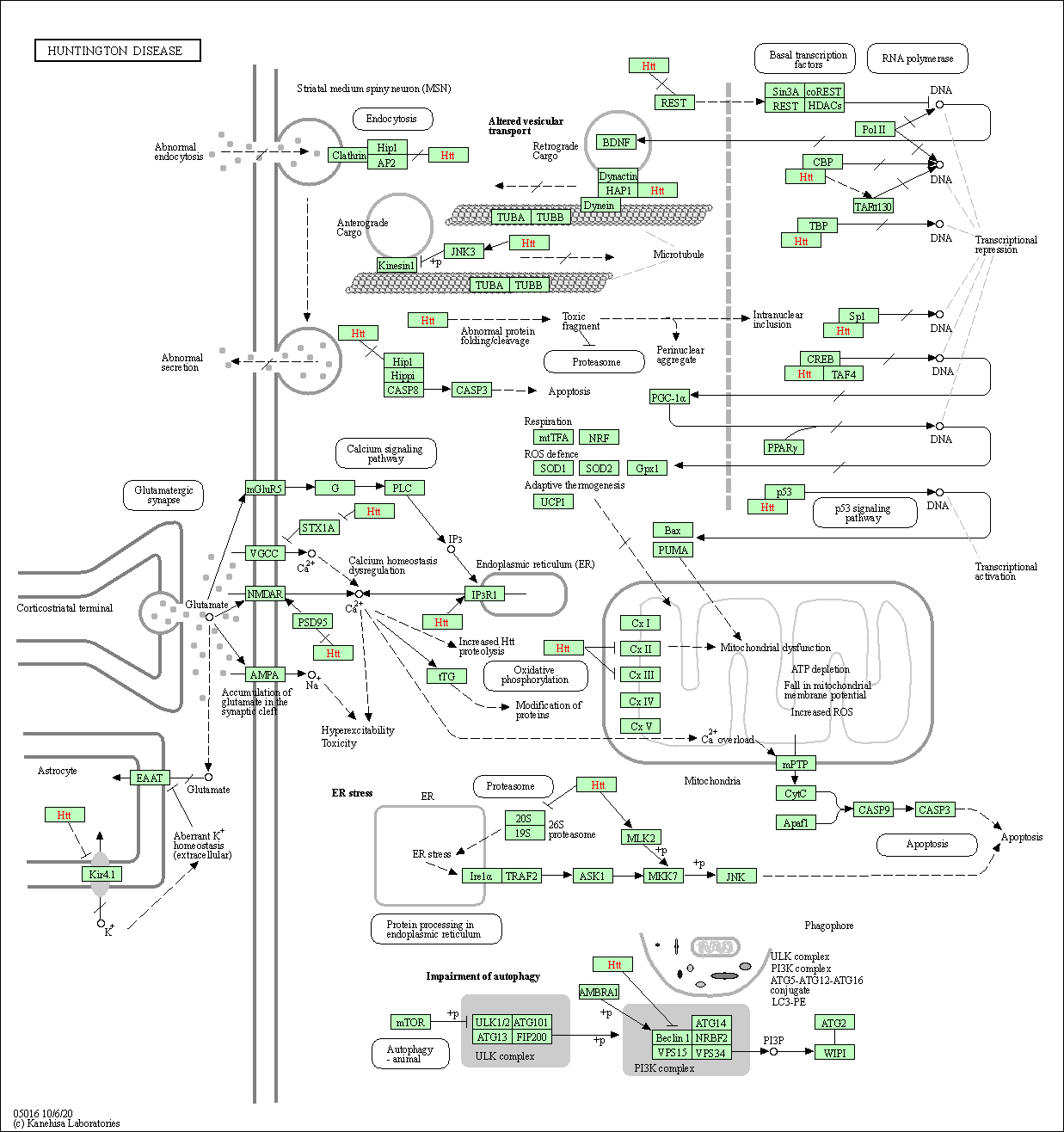
**

**Figure S5 KEGG’S pathways shows contributed genes in pathways related to Huntington’s disease**
